# Synthesis and Biological Evaluation of a Series of N-Substituted Pyruvamide 4-Allylthiosemicarbazones and Their Copper(II) Complexes: Antibacterial, Antiradical, and Antiproliferative Activities

**DOI:** 10.3390/molecules31162857

**Published:** 2026-08-16

**Authors:** Ianina Graur, Dorin Istrati, Vasilii Graur, Victor Tsapkov, Olga Garbuz, Elena Melnic, Pavlina Bourosh, Jenny Roy, Aurelian Gulea

**Affiliations:** 1 Laboratory of Advanced Materials in Biopharmaceutics and Technics, Institute of Chemistry, Moldova State University, MD-2009 Chișinău, Moldova; ulchina.ianina@usm.md (I.G.); vtsapkov@gmail.com (V.T.); 2 Department of Dentistry, University of Medicine and Pharmacy “Nicolae Testemitanu”, MD-2004 Chisinau, Moldova; dorin.istrati@usmf.md; 3 Laboratory of Systematics and Molecular Phylogenetics, Institute of Zoology, Moldova State University, 1 Academiei Street, MD-2028 Chișinău, Moldova; olga.garbuz@sti.usm.md; 4 Laboratory of Physical Methods of Solid State Investigation “Tadeusz Malinowski”, Institute of Applied Physics, Moldova State University, MD-2028 Chișinău, Moldova; elena.melnic2@gmail.com (E.M.); bourosh.xray@gmail.com (P.B.); 5 Department of Molecular Medicine, Faculty of Medicine, Université Laval, Québec, QC G1V 0A6, Canada; jenny.roy@crchudequebec.ulaval.ca

**Keywords:** thiosemicarbazone, pyruvamide, copper(II) complexes, antiproliferative activity, antibacterial activity, antioxidant activity

## Abstract

A series of pyruvamide 4-allylthiosemicarbazones **HL^1^**^–**6**^ and their copper(II) chloride complexes **1**–**6** were synthesized. Their composition and structures were studied using elemental analysis, ^1^H and ^13^C NMR spectroscopy, and FTIR spectroscopy, as well as single-crystal X-ray diffraction analysis for thiosemicarbazones **HL^2^**, **HL^4^**, and **HL^6^**. The antiproliferative activity was evaluated against three human cancer cell lines. The copper(II) complexes exhibit higher anticancer activity than the corresponding thiosemicarbazones. Complexes **1** and **3** demonstrate the most promising anticancer properties and, in some cases, surpass the activity of doxorubicin, the reference drug. Their selectivity indices toward human leukemia THP-1 cells relative to the normal hTERT-RPE1 cell line are greater than 10. The copper(II) coordination compounds also exhibit moderate antibacterial and antifungal properties; however, this activity does not exceed that of the standard drugs. In contrast, thiosemicarbazones **HL^1^**^–**6**^ show high antiradical activity, which surpasses that of both the corresponding copper(II) complexes and Trolox. All studied biological activities strongly depend on the nature of the substituent at the nitrogen atom of the pyruvamide moiety, highlighting the potential for further exploration of substituted pyruvamides with improved activity.

## 1. Introduction

One of the key aims of modern medicinal chemistry is the discovery and development of new bioactive compounds with improved therapeutic potential and diverse biological effects. Thiosemicarbazones represent an important class of compounds in pharmaceutical research, as they serve as both versatile synthetic intermediates and effective chelating agents capable of forming stable complexes with transition metals. Numerous studies have demonstrated that thiosemicarbazones and their metal complexes exhibit significant pharmacological properties, often showing enhanced efficacy and reduced toxicity compared to conventional agents [1,2,3].

The biological activity of thiosemicarbazones is mainly attributed to their interference with DNA-related processes. Proposed mechanisms include inhibition of DNA synthesis through suppression of ribonucleotide diphosphate reductase, interactions with nucleic acid bases that obstruct replication, and induction of oxidative damage leading to DNA strand disruption [4,5]. Furthermore, extensive research indicates that the biological performance of thiosemicarbazones is strongly influenced by their ability to coordinate metal ions. In many cases, metal complexation significantly enhances biological activity, highlighting the crucial role of the metal center. Coordination can modify the lipophilicity of the ligand, facilitating cellular uptake, and may also reduce undesirable side effects observed for free ligands [6,7]. Notably, metal complexes often display novel or amplified biological effects that are absent in the uncoordinated thiosemicarbazones, underscoring their potential as promising therapeutic candidates.

Since the 1970s, following the introduction of cisplatin as the first successful platinum-based anticancer drug, research interest in metal-containing therapeutic agents has increased markedly. Despite its clinical efficacy, the application of platinum compounds is considerably restricted due to severe adverse effects, including nephrotoxicity, neurotoxicity, ototoxicity, and myelosuppression. These limitations have stimulated the search for alternative metal-based systems that are better tolerated by the human organism [8].

As a result, compounds incorporating essential or biocompatible metals such as zinc, copper(II), and iron(III) have attracted growing attention [9]. Among them, copper(II) has emerged as one of the most intensively studied metals in anticancer research [10,11,12]. Numerous investigations have demonstrated that copper(II) complexes can effectively induce cancer cell death through multiple mechanisms, including inhibition of the proteasome, generation of reactive oxygen species, and induction of DNA damage [13,14,15]. An additional advantage of copper-based compounds lies in the presence of endogenous biological pathways responsible for copper homeostasis and detoxification, which may contribute to improved tolerance compared to platinum-based drugs. Consequently, copper(II) complexes are increasingly regarded as promising alternatives to traditional platinum therapeutics in anticancer drug development.

Thiosemicarbazone copper(II) complexes have attracted considerable attention due to their pronounced antibacterial activity. Coordination of thiosemicarbazone ligands to Cu(II) ions often enhances their biological efficacy compared to the free ligands, which is commonly attributed to increased lipophilicity and improved penetration through bacterial cell membranes [16,17,18,19]. These complexes can interact with key cellular targets, induce oxidative stress via reactive oxygen species generation, and disrupt essential enzymatic processes in bacteria. As a result, copper(II) thiosemicarbazone complexes frequently show higher activity against Gram-positive bacteria and represent promising candidates for the development of new antibacterial agents, especially in the context of increasing antimicrobial resistance [20].

Thiosemicarbazone copper(II) complexes have attracted significant interest as potential antioxidant agents due to the redox-active nature of the Cu(II)/Cu(I) couple. Coordination of thiosemicarbazone ligands to copper ions can modulate electron-transfer properties, enhance radical-scavenging ability, and influence interactions with reactive oxygen species [21,22]. In many cases, copper(II) complexes exhibit higher antioxidant activity than the corresponding free ligands, which is attributed to increased stability and improved redox behavior upon complexation [23,24]. These properties make thiosemicarbazone–Cu(II) complexes promising candidates for further investigation as metal-based antioxidants with potential biomedical applications.

**Figure 1:**
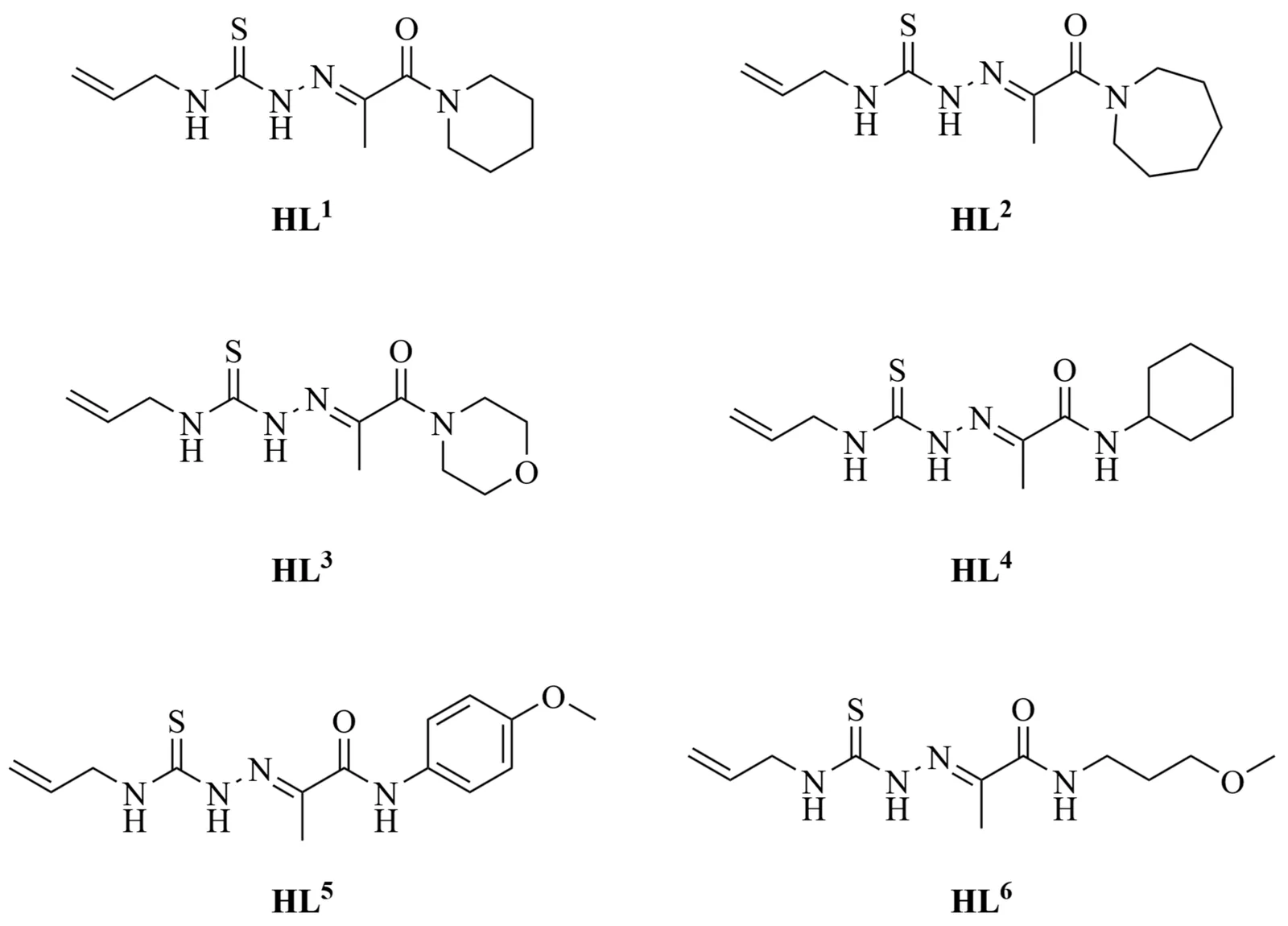
The structural formula of **HL^1^**^–**6**^.

Previously, we have published studies on the properties of 1-(piperidin-1-yl)propane-1,2-dione 4-allylthiosemicarbazone [25] and 3-(morpholin-4-yl)propane-2,3-dione 4-allylthiosemicarbazone [26] and their antibacterial, antifungal and antioxidant activities. The aim of this work was the synthesis of various pyruvamide 4-allylthiosemicarbazones (Figure 1) and their copper(II) chloride complexes, as well as the investigation of their antiproliferative, antibacterial, antifungal, and antioxidant activities.

## 2. Results and Discussion

### 2.1. Synthesis and Characterization

**Figure 2:**
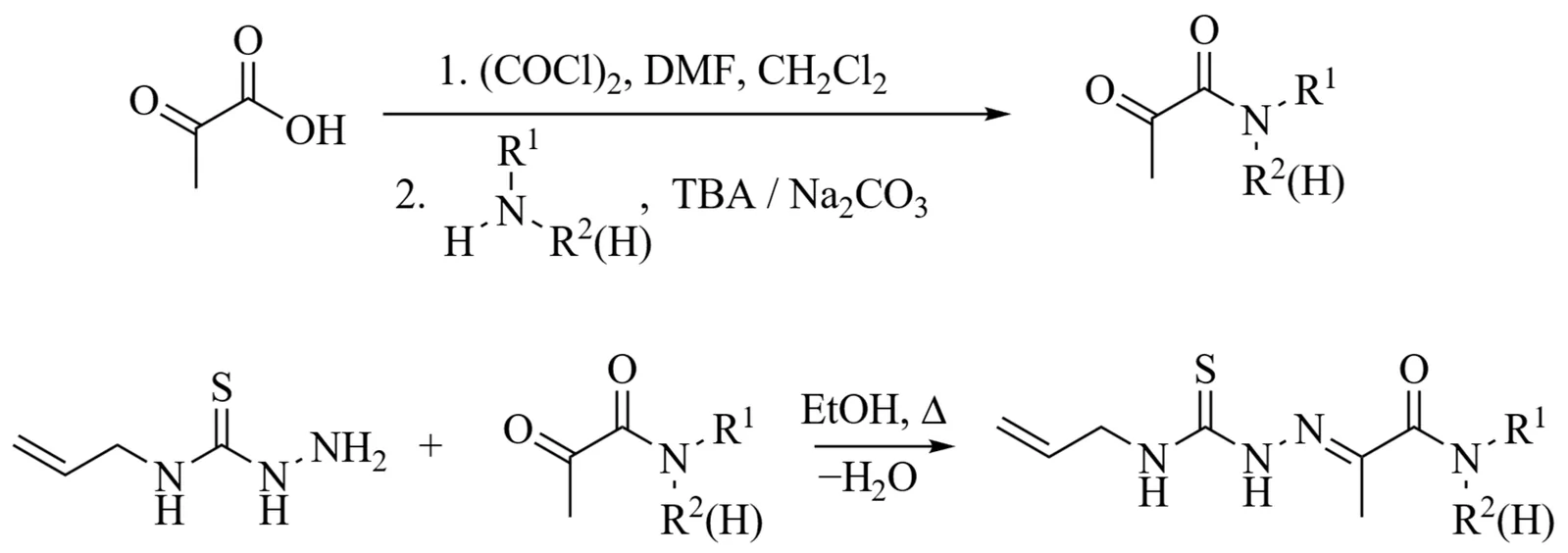
Synthesis of pyruvamide 4-allylthiosemicarbazones **HL^1^**^–**6**^.

A series of six pyruvamide-based 4-allylthiosemicarbazones (**HL^1^**–**HL^6^**) was synthesized through a two-step procedure (Figure 2). Initially, the corresponding pyruvamide derivatives were prepared via acylation of the appropriate amines with pyruvic acid activated by oxalyl chloride in dichloromethane. Subsequently, condensation of the obtained pyruvamide derivatives with 4-allylthiosemicarbazide was carried out in ethanol using equimolar amounts of both reactants, affording the target thiosemicarbazones. The formation and structural identity of **HL^1^**–**HL^6^** were confirmed by ^1^H and ^13^C NMR spectroscopy (Figures S1–S12). Single crystals suitable for X-ray diffraction analysis were obtained for **HL^2^**, **HL^4^**, and **HL^6^** by slow recrystallization from ethanol. The syntheses of **HL^1^** [25] and **HL^3^** [26], as well as their structures (CCDC 2255382 and 2350142, respectively), have been previously reported.

**Figure 3:**
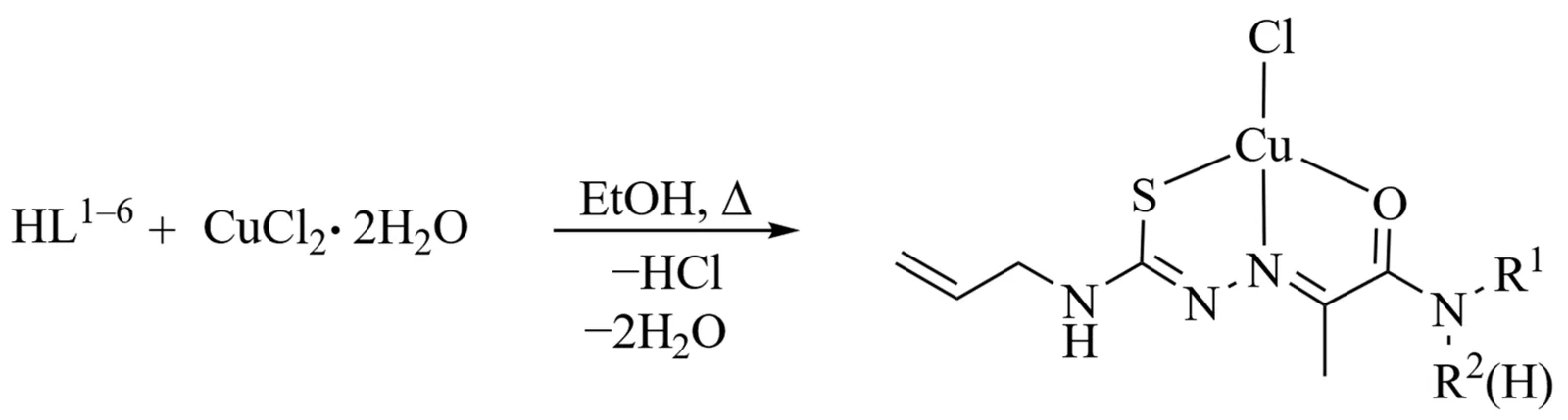
General scheme of the synthesis of copper(II) chloride complexes **1**–**6**.

Copper(II) coordination compounds **1**–**6** were prepared by treating the corresponding thiosemicarbazone ligands **HL^1^**–**HL^6^** with copper(II) chloride dihydrate in ethanol using an equimolar ligand-to-metal ratio (Figure 3). Elemental analysis data were consistent with the proposed composition of the complexes, which can be described by the general formula [Cu(L^1–6^)Cl] (**1**–**6**). The molar conductivity measurements of complexes **1**–**6**, performed in methanol solution, gave values in the range of 70–95 Ω^−1^·cm^2^·mol^−1^, suggesting their electrolytic behavior as 1:1 electrolytes.

The syntheses of complexes **1** [25] and **3** [26] have been previously reported. Moreover, the crystal structure of complex **1** has been previously determined by single-crystal X-ray diffraction analysis (CCDC 2255383) [25]. It was shown that thiosemicarbazone HL^1^ acts as a monodeprotonated tridentate ligand with an ONS-set of donor atoms. Upon complexation, the thiosemicarbazone ligand forms two fused five-membered metallacycles. The copper(II) central atom in this complex has a coordination number of 4 and adopts a distorted square-planar geometry.

### 2.2. Spectroscopic Studies

The coordination behavior of the 4-allylthiosemicarbazone ligands **HL^1^**–**HL^6^** toward copper(II) ions was investigated by comparing the FTIR spectra of the free ligands and the corresponding complexes **1**–**6** (Figures S13–S24). Formation of the complexes is accompanied by the loss of one NH-related absorption band, which indicates deprotonation of the ligand during coordination. The bands associated with the remaining NH groups undergo shifts toward lower wavenumbers, suggesting their involvement in the coordination process.

Furthermore, coordination-induced changes were observed for the characteristic vibrations of the C=O and C=N groups, which were shifted to lower frequencies upon complex formation. A significant modification of the thioamide region was also detected: the bands assigned to ν(C=S) vibrations disappeared, while a new absorption band attributed to ν(C–S) appeared in the spectra of complexes **1**–**6**. These changes support the thiol–thione tautomeric transformation of the ligands and confirm coordination of the deprotonated thiolate form to the copper(II) center.

### 2.3. Structure Description of Thiosemicarbazones ***HL**^**2**,**4**,**6**^*

A single-crystal X-ray diffraction study indicates that **HL^2^** and **HL^6^·0.5H_2_O** crystallize in the triclinic *P*$\bar{1}$ space group, and the thiosemicarbazone **HL^4^** crystallizes in the monoclinic *P*2_1_/*c* space group (Table S2).

**Figure 4:**
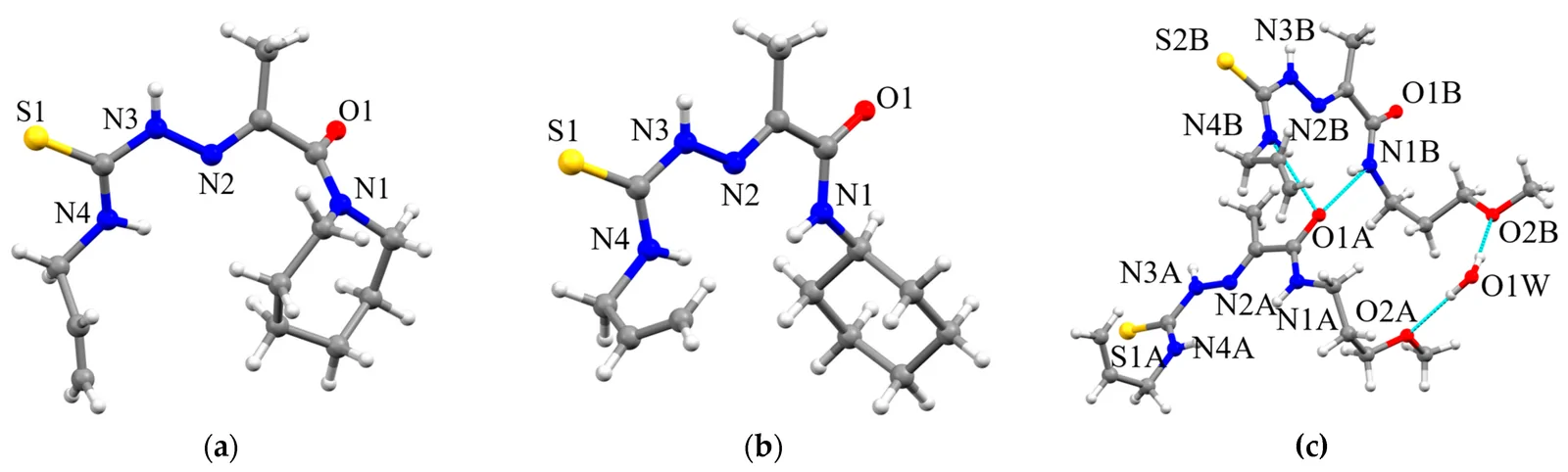
Molecular structures of compounds **HL^2^** (**a**)**_,_ HL^4^** (**b**) and **HL^6^·0.5H_2_O** (**c**) with partial atom numbering schemes.

The asymmetric units of the **HL^2^** and **HL^4^** ligands contain only one organic molecule, while for the thiosemicarbazone **HL^6^**, two crystallographically independent molecules and one molecule of crystallization water were established (Figure 4). Consequently, the formula for the latter compound is **HL^6^·0.5H_2_O**. Structural analysis shows that the thiosemicarbazone fragment is nearly planar in all three ligands, with a maximum deviation of 0.043 Å, and adopts an *E* configuration. This configuration is stabilized by N(4)–H···N(2) in **HL^2^** and by two N(4)–H···N(2) and N(1)–H···N(2) intramolecular hydrogen bonds in **HL^4^** and **HL^6^**·0.5H_2_O ligands; bond lengths are listed in Table S1. The C–N and C–S bond lengths in these fragments are indicative of the stabilization of the thione form (Table S3, Figure S25). The dihedral angles between the mean planes of the thiosemicarbazone fragment and the terminal azepane ring, cyclohexyl ring, and 3-methoxypropyl fragments in these compounds are 29.76^°^, 69.95^°^ and 78.10/78.05^°^ respectively.

**Figure 5:**
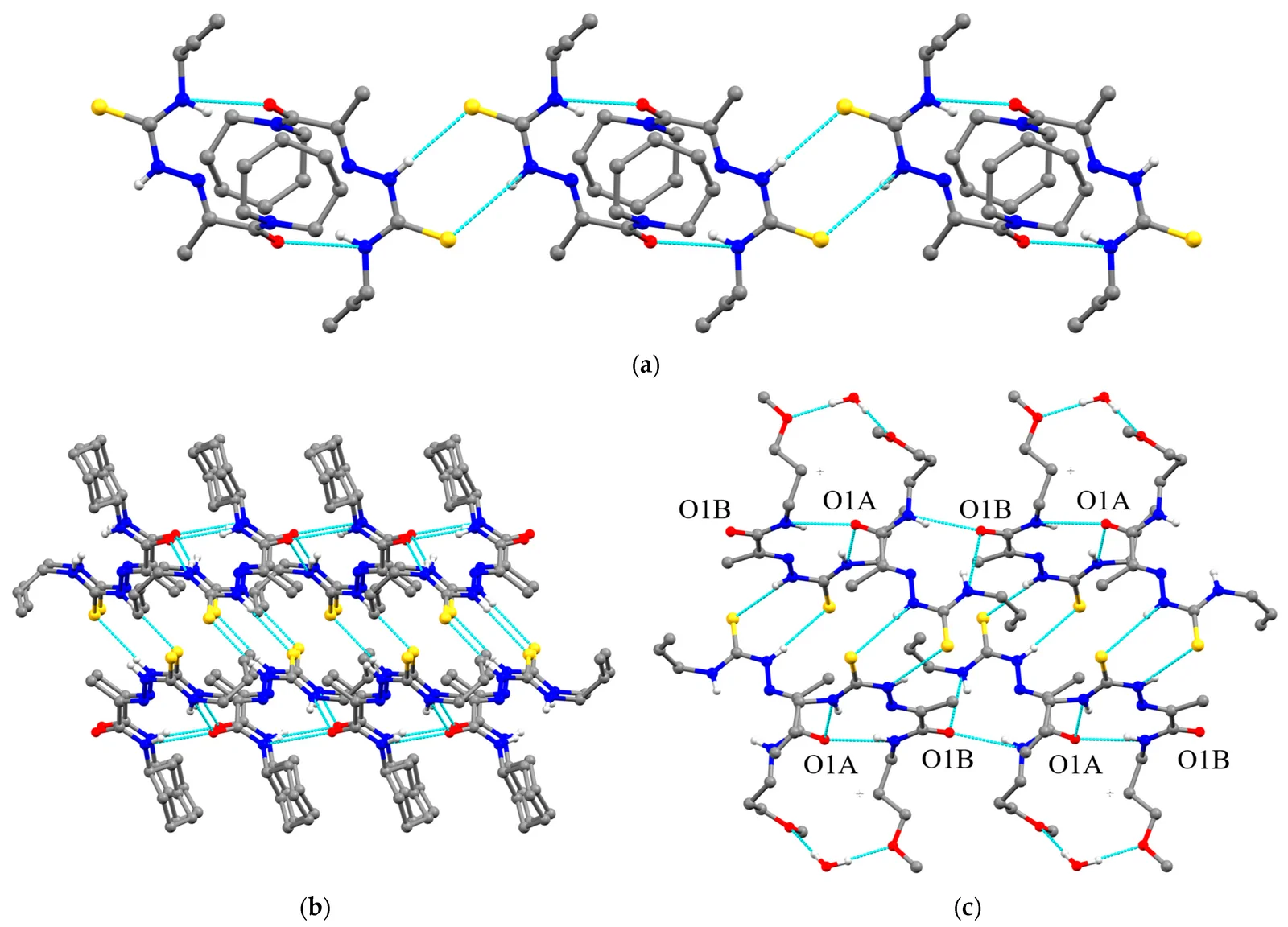
Supramolecular chain in the crystal structures of **HL**^2^ (**a**); supramolecular layers in the crystal structures of **HL^4^** (**b**) and **HL^6^·0.5H_2_O** (**c**).

In the **HL^2^** crystal structure, inversion-related molecules are connected by intermolecular N–H···S hydrogen bonds of synthon *R*^2^_2_(8) (Table S1), forming a supramolecular dimer. These dimers extend into supramolecular chains via N–H···O hydrogen bonds (Figure 5a). Similar to **HL^2^**, centrosymmetric dimers are formed in the crystal structures of **HL^4^** and **HL^6^**·0.5H_2_O, but their crystal packing differs. The distinct arrangement of organic molecules in the resulting supramolecular layers of **HL^4^** and **HL^6^**·0.5H_2_O can be explained by the presence of an additional N(1)-H donor group involved in intermolecular hydrogen bonding in both compounds, as well as the presence of water of crystallization in the latter (Figure 5b,c and Table S1).

### 2.4. Anticancer Activity

**Figure 6:**
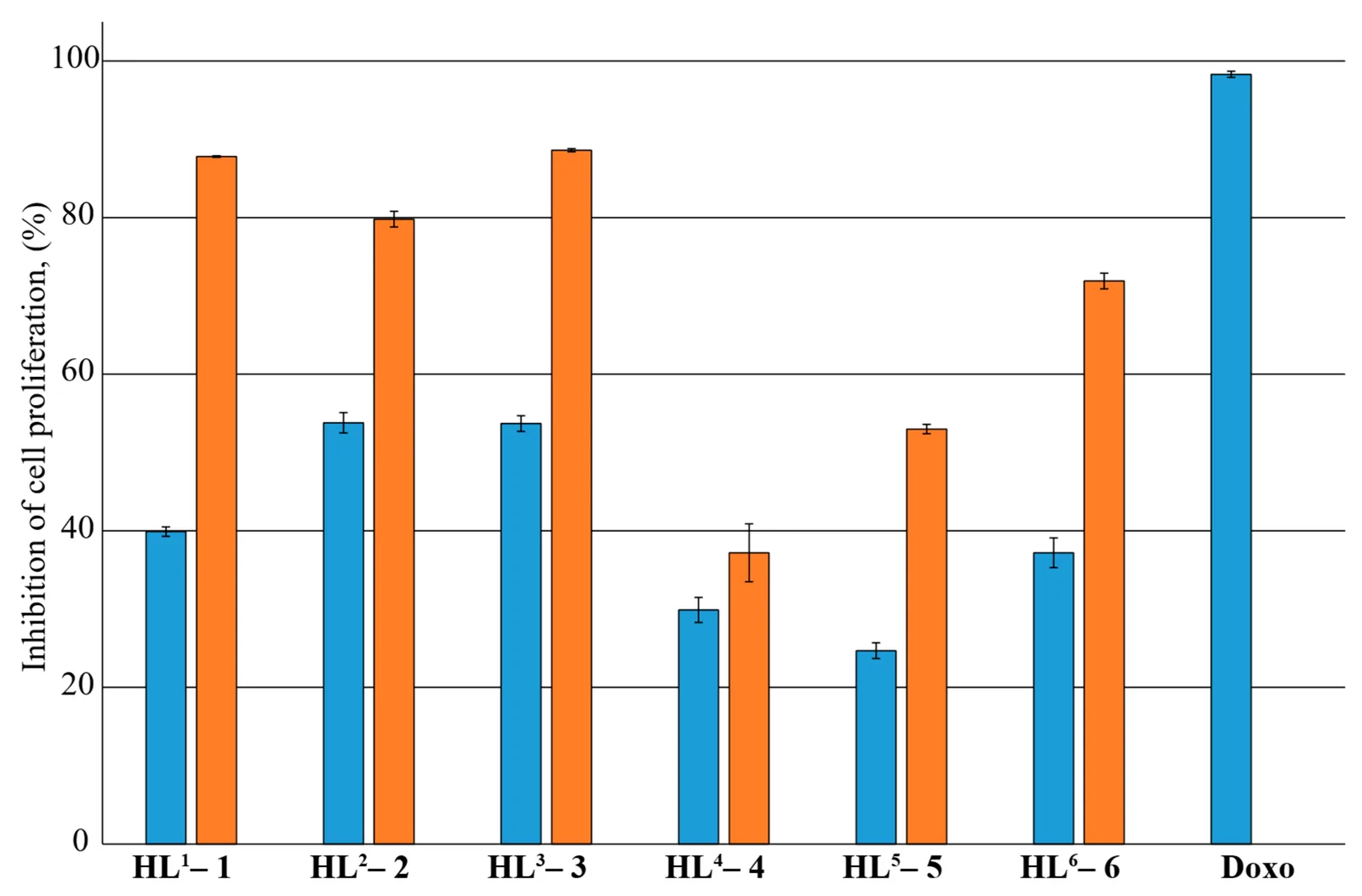
Antiproliferative activity of the synthesized thiosemicarbazones and their copper(II) complexes against the human leukemia THP-1 cell line at 10 µM.

The antiproliferative properties of the investigated compounds were evaluated using the human leukemia THP-1 cell line. The cells were exposed to the tested substances at concentrations of 10, 1, and 0.1 µM, and the inhibitory effects on cell proliferation were determined. The percentage of inhibition at a concentration of 10 µM is shown in Figure 6.

The antiproliferative activity of the thiosemicarbazones depends on the nature of the substituent at the nitrogen atom of the pyruvamide fragment. When comparing the percentage of inhibition of cell proliferation at a concentration of 10 µM, the activity increases in the following order: **HL^5^** < **HL^4^** < **HL^6^**·0.5H_2_O < **HL^1^** < **HL^2^** ≈ **HL^3^**. The latter two compounds inhibit cell proliferation by more than 50%; therefore, their IC_50_ values were calculated: 3.85 ± 0.36 µM (**HL^2^**) and 2.79 ± 0.28 µM (**HL^3^**).

The obtained results, presented as the percentage of proliferation inhibition at each tested concentration, together with the calculated half-maximal inhibitory concentration (IC_50_) values, are summarized in Table 1.

As can be seen from the obtained data, in all cases copper(II) coordination compounds exhibit higher antiproliferative activity than their corresponding ligands against this cancer cell line. All complexes, except complex **4**, inhibit THP-1 cell proliferation by more than 50% at a concentration of 10 µM, which allows calculation of their IC_50_ values. Based on the IC_50_ values, the activity of the complexes increases in the following order: **4** < **5** < **1** < **6** < **2** < **3**. Thus, complexes **2** and **3**, as well as their corresponding thiosemicarbazones **HL^2^** and **HL^3^** containing the moieties of hexamethyleneimine and morpholine in their pyruvamide moiety, are the most active compounds from the studied series of substances. Notably, complex **3** (IC_50_ 0.14 ± 0.01 µM) is 2.3-fold more active than doxorubicin (IC_50_ 0.32 ± 0.06 µM), which was used as a reference drug in this study to compare the antiproliferative activity.

**Figure 7:**
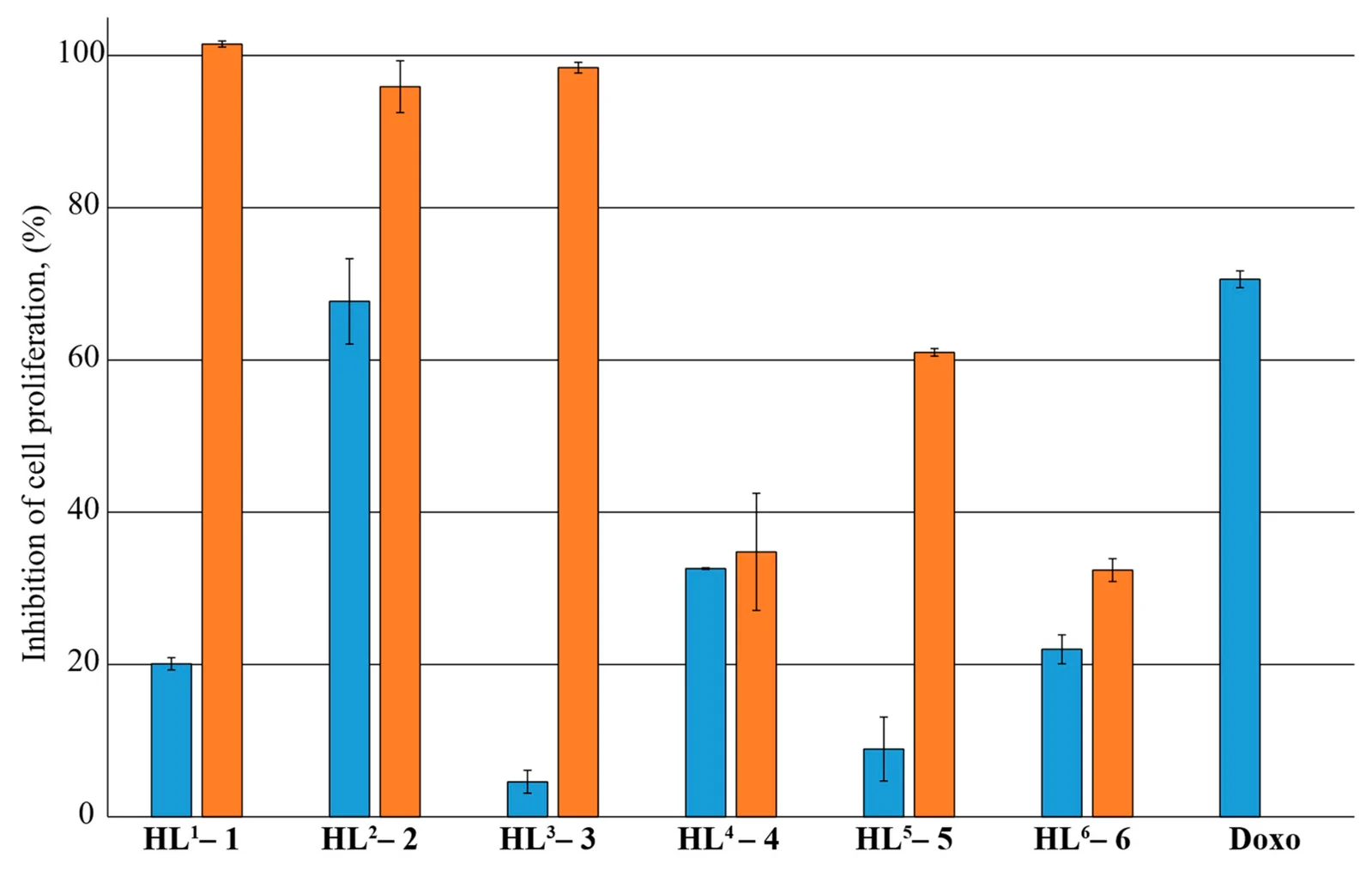
Antiproliferative activity of the synthesized thiosemicarbazones and their copper(II) complexes against the human prostate cancer PC-3 cell line at 10 µM.

The antiproliferative activity was also evaluated against the human prostate cancer PC-3 cell line. In general, PC-3 cells were less sensitive to the studied series of substances. The percentage of inhibition at a concentration of 10 µM is shown in Figure 7.

As was observed for the THP-1 cell line, the copper(II) complexes exhibit higher antiproliferative activity against PC-3 cells than the corresponding thiosemicarbazones. Only five of the studied substances inhibit proliferation of PC-3 cells by more than 50% at 10 µM: thiosemicarbazone **HL^2^** and complexes **1**, **2**, **3**, and **5**. The inhibition percentages at three concentrations and the calculated IC_50_ values for these compounds are summarized in Table 2.

Coordination compound **1** demonstrated the highest antiproliferative activity among the tested compounds toward the PC-3 cell line. Nevertheless, its activity was approximately two times lower than that of doxorubicin, which showed an IC_50_ value of 0.83 ± 0.66 µM.

**Figure 8:**
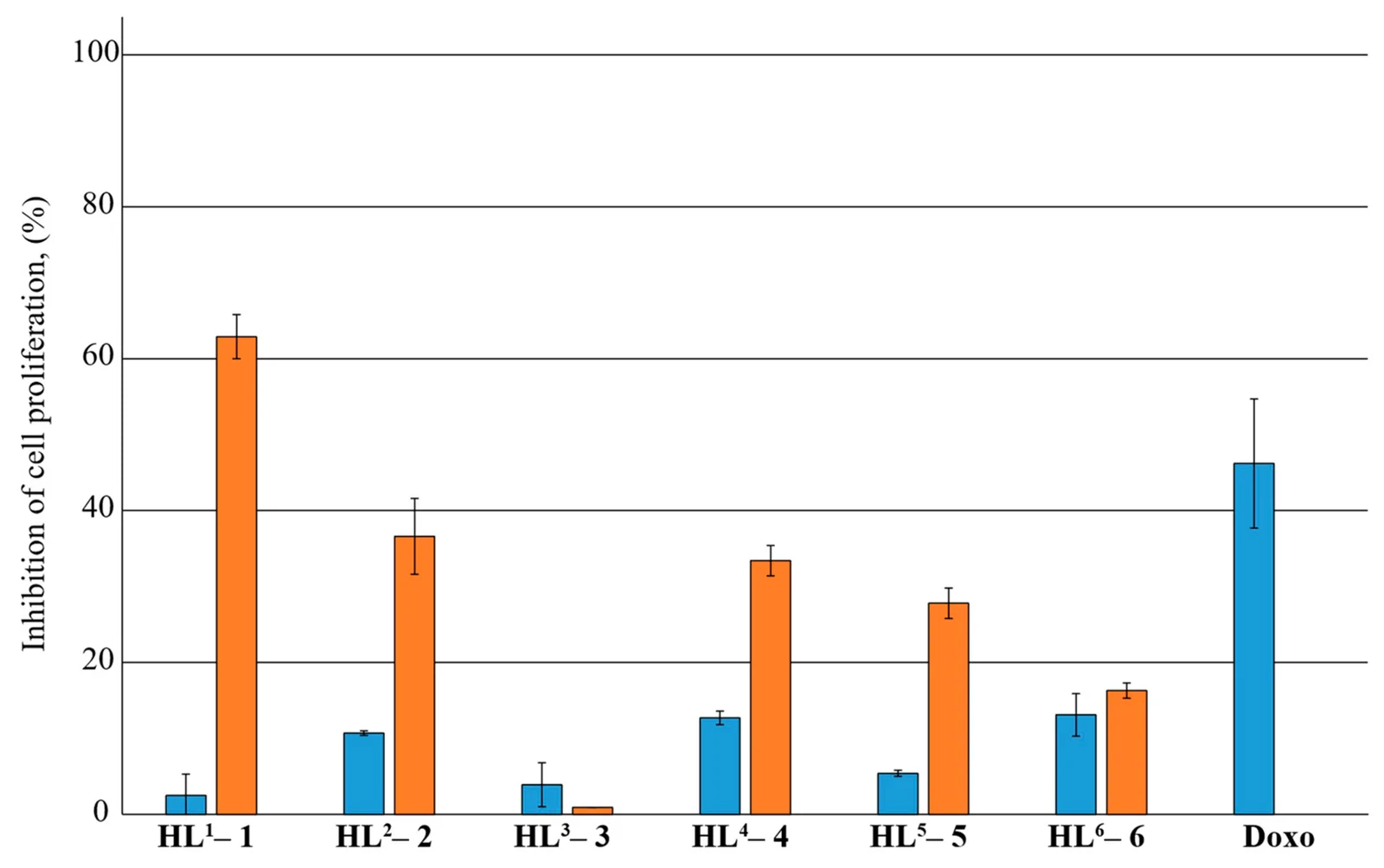
Antiproliferative activity of the synthesized thiosemicarbazones and their copper(II) complexes against the human colorectal cancer HT-29 cell line at 10 µM.

The antiproliferative effects of the investigated compounds were subsequently assessed using the human colorectal cancer HT-29 cell line. The inhibition rates determined at a concentration of 10 µM are presented in Figure 8.

Among the investigated compounds, only complex **1** was able to reduce the proliferation of the HT-29 cell line by more than 50%, showing a greater effect than doxorubicin under the tested conditions. The inhibition percentages at three concentrations and the calculated IC_50_ values for these compounds are summarized in Table 3. The IC_50_ value determined for complex **1** against HT-29 cells, based on the inhibition data obtained at three concentrations (10 µM: 62.9 ± 2.9%; 1 µM: 4.3 ± 2.8%; 0.1 µM: 1.8 ± 0.9%), is 7.20 ± 0.02 µM. This value is approximately three times lower than that obtained for doxorubicin (IC_50_ = 23.7 ± 5.1 µM), for which the corresponding inhibition values were 62.6 ± 3.7% at 100 µM, 46.2 ± 8.5% at 10 µM, and 14.6 ± 5.7% at 1 µM.

Considering that complexes **1** and **3**, incorporating piperidine and morpholine substituents in the pyruvamide moiety, exhibited the most pronounced antiproliferative effects, their selectivity toward cancer cells was further examined using the normal hTERT-RPE1 cell line as a reference. For complex **1**, the inhibition of cell proliferation was 42.6 ± 0.5% and 38.6 ± 0.1% at concentrations of 10 and 1 µM, respectively. In the case of complex **3**, higher inhibition was observed at 10 µM (71.2 ± 1.3%), whereas the inhibitory effect decreased to 6.2 ± 0.4% at 1 µM.

Complex **3** demonstrated a pronounced inhibitory effect on the proliferation of the normal hTERT-RPE1 cell line, reducing cell viability by more than 50% at a concentration of 10 µM. This indicates relatively high cytotoxicity toward normal cells, which is reflected by its IC_50_ value of 5.59 ± 0.23 µM. Accordingly, the calculated selectivity indices (SI), determined as the ratio between the IC_50_ values obtained for the normal and cancer cell lines, were 40 for THP-1 and 1.9 for PC-3 cells.

In comparison, complex 1 exhibited considerably lower toxicity toward normal cells, with an IC_50_ value above 10 µM. The corresponding selectivity indices were SI(THP-1) > 10, SI(PC-3) > 6, and SI(HT-29) > 1.3.

Doxorubicin showed the lowest cytotoxicity toward the normal cell line among the tested compounds, with an IC_50_ value of 55.5 ± 4.9 µM. Its selectivity indices were calculated as SI(THP-1) = 170, SI(PC-3) = 66, and SI(HT-29) = 2.3.

### 2.5. Antibacterial and Antifungal Activities

The antibacterial and antifungal activities of **HL^1^**^–**5**^ and their copper(II) complexes **1**–**5** were evaluated against Gram-positive bacteria (*S. aureus* and *B. cereus*) and the fungal strain *C. albicans*. The results are presented in Table 4 as minimum inhibitory, bactericidal, and fungicidal concentrations (MIC, MBC, and MFC, respectively). In almost all cases, coordination of the ligands to the copper(II) center resulted in an enhancement of antibacterial and antifungal activity compared with the corresponding free ligands, indicating that metal coordination plays an important role in modulating the antimicrobial properties of these compounds. This effect may be associated with changes in the electronic properties, lipophilicity, and overall molecular structure of the ligands upon coordination, which can facilitate their interaction with microbial cells.

Among the investigated compounds, complex **5** exhibited the most pronounced antimicrobial activity, particularly against *Bacillus cereus*, indicating that this complex may represent the most promising compound of the series. In general, the synthesized compounds showed higher activity toward Gram-positive bacteria than toward *C. albicans*, suggesting that the nature of the microbial cell envelope may influence their susceptibility to the tested compounds. The antibacterial activity of the copper(II) complexes followed the order **5** > **4** > **1** > **2** > **3**. This trend demonstrates that relatively small structural modifications within the ligand series can substantially affect the biological activity of the resulting copper(II) complexes.

Overall, the obtained results demonstrate that copper(II) coordination can significantly enhance the antimicrobial properties of the thiosemicarbazone ligands. The particularly high activity of complex **5** against *B. cereus* highlights this compound as a promising candidate for further investigation and suggests that the structural features of the ligand play an important role in determining the antimicrobial activity of its copper(II) complex.

### 2.6. Antioxidant Activity

The antioxidant potential of 4-allylthiosemicarbazones **HL^1^**^–**6**^ and their copper(II) coordination complexes **1**–**6** was evaluated using the ABTS^•+^ radical cation assay. The assessment of antioxidant activity was included as part of the broader biological characterization of the synthesized compounds because oxidative stress and the imbalance between reactive oxygen species and cellular antioxidant defenses are closely associated with a number of pathological processes, including inflammation, microbial infections, and cancer. Therefore, evaluation of the radical-scavenging properties provides complementary information on the biological profile of the synthesized thiosemicarbazones and their copper(II) complexes. In addition, comparison of antioxidant, antimicrobial, and antiproliferative activities may help to identify whether the biological effects of structural modifications and metal coordination follow similar or distinct trends.

The activity was quantified by determining the half-maximal inhibitory concentration (IC_50_) values, which are summarized in Table 5. The obtained results reveal a consistent trend observed for all synthesized compounds: the copper(II) complexes exhibit lower antioxidant activity than the corresponding ligands from which they were obtained. This indicates that, in contrast to the antibacterial activity, coordination of the 4-allylthiosemicarbazones to the copper(II) center does not enhance their radical-scavenging ability under the experimental conditions used.

Nevertheless, several ligands and their copper(II) complexes, namely **HL^2^** and **HL^6^·0.5H_2_O** as well as complexes **2** and **6**, demonstrated higher activity than Trolox, the standard antioxidant used for comparison. The antioxidant activity of the ligands decreased in the following order: **HL^2^** > **HL^6^·0.5H_2_O** > **HL^1^** > **HL^5^** > **HL^4^** > **HL^3^**, whereas the activity of the complexes followed the order **2** > **6** > **5** > **1** > **3** > **4**. Among all synthesized compounds, **HL^2^** exhibited the highest antioxidant activity, with an IC_50_ value of 3.85 ± 0.18 μM.

The differences observed in antioxidant activity indicate that the structural characteristics of the ligands play an important role in determining their radical-scavenging properties. In particular, the markedly higher activity of **HL^2^** compared with the other ligands suggests that the nature and arrangement of the substituents influence the ability of the molecule to interact with and neutralize the ABTS^•+^ radical cation. The decrease in antioxidant activity observed upon copper(II) coordination further indicates that metal coordination can modify the availability and reactivity of functional groups involved in radical scavenging.

When considered together with the antimicrobial and antiproliferative results, the antioxidant data demonstrate that the biological effects of the synthesized compounds do not follow a single common trend. Copper(II) coordination generally enhanced the antimicrobial activity of the ligands, whereas it decreased their antioxidant activity. This difference suggests that the enhanced antimicrobial properties of the copper(II) complexes cannot be attributed simply to their radical-scavenging capacity. Similarly, the antioxidant activity should not be interpreted as a direct predictor of antiproliferative activity, since cellular responses depend on multiple factors, including cellular uptake, metal ion homeostasis, interactions with biomolecular targets, and the ability of the compounds to modulate intracellular redox processes.

Thus, the ABTS^•+^ results provide complementary information that helps distinguish the radical-scavenging properties of the compounds from their antimicrobial and antiproliferative effects [27]. The absence of a direct correlation between these activities is itself informative, indicating that different mechanisms may contribute to the biological effects of the investigated 4-allylthiosemicarbazones and their copper(II) complexes. Overall, **HL^2^** represents the most potent radical scavenger of the investigated series, while copper(II) coordination appears to favor antimicrobial activity at the expense of ABTS^•+^ radical-scavenging capacity.

## 3. Materials and Methods

3-Isothiocyanatoprop-1-ene, hydrazine hydrate, pyruvic acid, oxalyl chloride, sodium carbonate, dichloromethane, dimethylformamide, cyclohexanamine, azepane, 3-methoxypropan-1-amine, piperidine, morpholine, 4-methoxyaniline, tributylamine, acetone, and copper(II) chloride dihydrate were purchased from Sigma-Aldrich, Merck Group (Darmstadt, Germany) and used without further purification.

The ^1^H and ^13^C NMR spectra were acquired on a Bruker DRX-400 spectrometer (Ettlingen, Germany) using CDCl_3_ as the solvent. FTIR measurements were carried out on a Bruker ALPHA FTIR spectrophotometer at room temperature within the 4000–400 cm^−1^ spectral range. The copper [28] and chlorine [29] contents were determined by a titrimetric method. The determination of C, H, N, and S content was performed using the automatic Perkin Elmer 2400 elemental analyzer (Waltham, MA, USA). The electrical resistance of methanolic solutions of the complexes (c = 0.001 M, 20 °C) was measured using an R-38 rheochord bridge. The purity of the synthesized compounds was estimated from the ^1^H NMR spectra by comparing the integrated areas of characteristic proton signals with their theoretical integral values expected for the proposed molecular structures.

4-Allylthiosemicarbazide was prepared according to the previously described method [30] by reacting allyl isothiocyanate with a 50–60% aqueous hydrazine solution. The obtained compound exhibited physicochemical characteristics consistent with the reported literature data.

### 3.1. Synthesis of 4-Allylthiosemicarbazones ***HL^1^**^–**6**^*

General procedure for the synthesis of **HL^1^**^–**6**^

The synthesis of pyruvamide derivatives was carried out through the generation of 2-oxopropanoyl chloride followed by its reaction with the corresponding amines. Thus, pyruvic acid (8.80 g, 0.100 mol) was suspended in CH_2_Cl_2_ (5 mL), and a solution of oxalyl chloride (15.24 g, 0.120 mol) in CH_2_Cl_2_ (10 mL) was added at 0 °C under vigorous stirring. After the addition of several drops of DMF as a catalyst, the reaction vessel was equipped with a reflux condenser containing a calcium chloride drying tube. The mixture was subsequently heated with continuous stirring for approximately 1.5 h until formation of the corresponding acid chloride, observed as a yellow oily intermediate.

The freshly generated 2-oxopropanoyl chloride was then introduced dropwise into a cooled (0 °C) suspension containing the appropriate amine (0.100 mol), sodium carbonate (10.60 g, 0.100 mol) or tributylamine (18.5 g, 0.100 mol, for the reaction with 4-methoxyaniline), and CH_2_Cl_2_ (10 mL). The reaction mixture was allowed to warm to room temperature and stirred for an additional hour. After completion, the mixture was filtered, and the organic phase/residue was washed with water (3 × 100 mL). The obtained products were dried in air to give crude pyruvamide derivatives as yellow mobile oils. For the derivative obtained from 4-methoxyaniline, a light gray solid was isolated after filtration and further purified by recrystallization from acetone, affording a white crystalline product.

Subsequently, the synthesized pyruvamide derivative (20.0 mmol) was dissolved in ethanol (25 mL), followed by the addition of N-(prop-2-en-1-yl)hydrazinecarbothioamide (20.0 mmol). The mixture was heated under stirring for 1 h, after which the formed pale-yellow precipitate was collected by filtration, washed with a small amount of ethanol, and dried in air to obtain the target 4-allylthiosemicarbazone.

Single crystals of ligands **HL^2^**, **HL^4^**, and **HL^6^** suitable for X-ray diffraction analysis were obtained by recrystallization from a saturated ethanol solution. The saturated solutions were left to undergo slow solvent evaporation at room temperature. Suitable single crystals formed within 2–4 days and were collected for X-ray diffraction analysis.

3-(Piperidin-1-yl)propane-2,3-dione 4-allylthiosemicarbazone **HL^1^**

Pale-yellow solid. Yield: 83%; mp 137–139 °C; purity > 95%; FW: 268.38 g/mol; Anal. Calc. for C_12_H_20_N_4_OS: C, 53.70; H, 7.51; N, 20.88; S, 11.95; found: C, 53.60; H, 7.42; N, 20.79; S, 11.87%. ^1^H NMR (CDCl_3_, 400 MHz) 8.71 (br., 1H; NH); 7.46 (br., 1H; NH); 5.89 (m., 1H; CH); 5.20 (m., 2H; CH_2_); 4.30 (m., 2H; NH-CH_2_); 3.50 (m., 2H; CH_2piperidine_); 2.11 (s., 3H; CH_3_); 1.62 (m. 6H; CH_2piperidine_). ^13^C NMR (CDCl_3_, 100 MHz) 178.20 (C=S); 165.33 (C=O); 143.47 (C=N); 132.88 (CH=CH_2_), 117.37 (CH_2_=), 46.93 (C_allyl_); 45.54, 45.51, 26.02, 24.44, 20.35 (C_piperidine_); 14.19 (CH_3_). FTIR data (cm^–1^): 3315, 3288 (NH), 1632 (C=O), 1620 (C=N), 1362 (C=S). The characteristics of the obtained substance correspond to the data reported in the literature [20].

3-(Azepan-1-yl)propane-2,3-dione 4-allylthiosemicarbazone **HL^2^**

Pale-yellow solid. Yield: 78%; mp 106–107 °C; purity > 95%; FW: 282.40 g/mol; Anal. Calc. for C_13_H_22_N_4_OS: C, 55.29; H, 7.85; N, 19.84; S, 11.35; found: C, 55.20; H, 7.78; N, 19.76; S, 11.28%. ^1^H NMR (CDCl_3_, 400 MHz) 8.73 (br s, 1H; NH); 7.42 (br s, 1H; NH); 5.89 (m, 1H; CH); 5.22 (m, 2H; CH_2_=C); 4.30 (m, 2H; NH-CH_2_); 3.50 (t, 2×2H; CH_2(hexamethyleneimine)_-N); 2.11 (s, 3H; CH_3_); 1.8–1.5 (m, 4×2H; CH_2(hexamethyleneimine)_). ^13^C NMR (CDCl_3_, 100 MHz) 178.29 (C=S); 166.76 (C=O); 143.93 (C=N); 132.86 (CH=CH_2_); 117.44 (CH_2_=); 46.98 (C_allyl_); 47.76, 45.38, 28.30, 27.00, 20.34 (C_(hexamethyleneimine)_); 14.10 (CH_3_). FTIR data (cm^–1^): ν(N–H) 3286, 3166; ν (C=O) 1643; ν (C=N) 1624; ν (C=S) 1362.

3-(Morpholin-4-yl)propane-2,3-dione 4-allylthiosemicarbazone **HL^3^**

Pale-yellow solid. Yield: 78%; mp 138–139 °C; purity > 95%; FW: 270.35 g/mol; Anal. Calc. for C_11_H_18_N_4_O_2_S: C, 48.87; H, 6.71; N, 20.72; S, 11.86; found: C, 48.78; H, 6.63; N, 20.63; S, 11.78%. ^1^H NMR (CDCl_3_, 400 MHz) 8.75 (br s, 1H; NH); 7.39 (br s, 1H; NH); 5.89 (m, 1H; CH); 5.21 (m, 2H; CH_2_); 4.30 (m, 2H; NH–CH_2_); 3.76–3.56 (m, 4×2H; CH_2(morpholine)_); 2.11 (s, 3H; CH_3_). ^13^C NMR (CDCl_3_, 100 MHz) 178.30 (C=S); 165.51 (C=O); 142.32 (C=N); 132.83 (CH(_allyl)_); 117.50 (CH_2(allyl,sp_^2^_)_); 66.72 (CH_2_O_(morpholine)_); 46.95 (CH_2(allyl,sp_^3^_)_ and CH_2_N_(morpholine)_); 14.13 (CH_3_). FTIR data (cm^–1^): ν(NH) 3282, 3200; ν (C=O) 1636; ν (C=N) 1619; ν (C=S) 1360. The characteristics of the obtained substance correspond to the data reported in the literature [21].

*N*-cyclohexyl-2-oxopropanamide 4-allylthiosemicarbazone **HL^4^**

Pale-yellow solid. Yield: 80%; mp 193–195 °C; purity > 95%; FW: 282.40 g/mol; Anal. Calc. for C_13_H_22_N_4_OS: C, 55.29; H, 7.85; N, 19.84; S, 11.35; found: C, 55.20; H, 7.78; N, 19.76; S, 11.28%. ^1^H NMR (CDCl_3_, 400 MHz) 8.61 (br s, 1H, NH-CO); 7.49 (br s, 1H, NH); 6.64 (d, 1H, NH); 5.95 (m, 1H, CH_allyl_); 5.31–5.18 (m, 2H, CH_2_=C); 4.35 (t, 2H, CH_2_); 3.79 (m, 1H, CH_(Cy)_); 2.11 (s, 3H, CH_3_); 1.97–1.07 (m, 10H, 5×CH_2(Cy)_). ^13^C NMR (CDCl_3_, 100 MHz) 178.21 (C=S); 162.28 (C=O); 142.95 (C=N); 132.98 (CH=CH_2_); 117.30 (CH_2_=); 48.69 (CH-NH); 47.11 (CH_2_-NH); 33.00, 25.45, 24.89 (CH_2(Cy)_); 10.67 (CH_3_). FTIR data (cm^–1^): ν(N–H) 3329, 3288, 3176; ν (C=O) 1640; ν (C=N) 1614; ν (C=S) 1365.

*N*-(4-methoxyphenyl)-2-oxopropanamide 4-allylthiosemicarbazone **HL^5^**

Pale-yellow solid. Yield: 80%; mp 154–156 °C; purity > 95%; FW: 306.38 g/mol; Anal. Calc. for C_14_H_18_N_4_O_2_S: C, 54.88; H, 5.92; N, 18.29; S, 10.47; found: C, 54.80; H, 5.86; N, 18.21; S, 10.39%. ^1^H NMR (CDCl_3_, 400 MHz) 8.70 (br s, 1H; NH); 8.66 (br s, 1H; NH); 7.84 (br s, 1H; NH); 7.44 (d, 2H, CH_(aromatic)_); 6.86 (d, 2H, CH_(aromatic)_); 5.96 (m, 1H; CH_(allyl)_); 5.32–5.20 (m, 2H, CH_2_=C); 4.36 (t, 2H, CH_2_); 3.80 (s, 3H, OCH_3_); 2.13 (s, 3H, CH_3_). ^13^C NMR (CDCl_3_, 100 MHz) 178.03 (C=S); 161.34 (C=O); 156.85, 129.95, 122.45, 144.20 (C_(aromatic)_); 142.48 (C-CH_3_); 132.95 (CH_(allyl)_); 117.56 (CH_2_=); 55.53 (CH_3_O); 47.29 (CH_2_-CH); 10.42 (CH_3_-C). FTIR data (cm^–1^): ν(N–H) 3357, 3305, 3198; ν (C=O) 1644; ν (C=N) 1615; ν (C=S) 1361.

*N*-(3-methoxypropyl)-2-oxopropanamide 4-allylthiosemicarbazone **HL^6^·0.5H_2_O**

Pale-yellow solid. Yield: 80%; mp 94–93 °C; purity > 95%; FW: 281.37 g/mol; Anal. Calc. for C_11_H_20_N_4_O_2_S·0.5H_2_O: C, 46.96; H, 7.52; N, 19.91; S, 11.39; found: C, 48.42; H, 7.30; N, 20.48; S, 11.68%. ^1^H NMR (CDCl_3_, 400 MHz) 8.69 (br s, 1H, NH-CO); 7.67 (br s, 1H, NH); 7.60 (br s, 1H, NH); 5.92 (m, 1H, CH_allyl_); 5.26 (d, 1H, CH_2_=C, J=16.4 Hz); 5.20 (d, 1H, CH_2_=C, J=9.8 Hz); 4.35 (m, 2H, CH_2_); 3.51 (t, 2H, CH_2_); 3.42 (m, 2H, CH_2_); 3.31 (s, 3H, OCH_3_); 2.10 (s, 3H, CH_3_); 1.80 (qnt, 2H, CH_2_). ^13^C NMR (CDCl_3_, 100 MHz) 178.25 (C=S); 163.21 (C=O); 142.31 (C-CH_3_); 132.92 (CH_allyl_); 117.47 (CH_2_=); 72.32 (CH_2_-O); 58.89 (CH_3_-O); 47.21 (CH_2_-CH_allyl_); 38.72 (CH_2_-NH); 28.54 (CH_2_); 10.48 (CH_3_-C). FTIR data (cm^–1^): ν(N–H) 3319, 3270, 3160; ν (C=O) 1644; ν (C=N) 1618; ν (C=S) 1363.

### 3.2. Synthesis of Copper(II) Coordination Compounds ***1**–**6***

General procedure for synthesis

Copper(II) complexes **1**–**6** were obtained by combining equimolar ethanolic solutions of the corresponding 4-allylthiosemicarbazone ligand **HL^1^**–**HL^6^** (1.00 mmol) and copper(II) chloride dihydrate (1.00 mmol). The reaction mixture was maintained until precipitation of a green solid was observed. The precipitated complexes were collected by filtration, washed with a small amount of ethanol, and dried under ambient conditions.

[Cu(L^1^)Cl] (**1**): Green solid. Yield: 82%. Anal. Calc. for C_12_H_19_ClCuN_4_OS (366.37 g mol^−1^): C, 39.34; H, 5.23; Cl, 9.68; Cu, 17.34; N, 15.29; S, 8.75; found: C, 39.25; H, 5.17; Cl, 9.54; Cu, 17.28; N, 15.15; S, 8.66. χ(CH_3_OH): 95 Ω^−1^ cm^−2^ mol^−1^. Main FTIR peaks (cm^−1^): ν(NH) 3219, ν(C=O) 1621, ν(C=N) 1574, ν(C–S) 791.

[Cu(L^2^)Cl] (**2**): Green solid. Yield: 88%. Anal. Calc. for C_13_H_21_ClCuN_4_OS (380.40 g mol^−1^): C, 41.05; H, 5.56; Cl, 9.32; Cu, 16.71; N, 14.73; S, 8.43; found: C, 40.95; H, 5.44; Cl, 9.22; Cu, 16.61; N, 14.64; S, 8.35. χ(CH_3_OH): 94 Ω^−1^ cm^−2^ mol^−1^. Main FTIR peaks (cm^−1^): ν(NH) 3237, ν(C=O) 1626, ν(C=N) 1575, ν(C–S) 775.

[Cu(L^3^)Cl] (**3**): Green solid. Yield: 72%. Anal. Calc. for C_11_H_17_ClCuN_4_O_2_S (368.34 g mol^−1^): C, 35.87; H, 4.65; Cl, 9.63; Cu, 17.25; N, 15.21; S, 8.71; found: C, 35.77; H, 4.57; Cl, 9.54; Cu, 17.17; N, 15.12; S, 8.64. χ(CH_3_OH): 79 Ω^−1^ cm^−2^ mol^−1^. Main FTIR peaks (cm^−1^): ν(NH) 3310, ν(C=O) 1625, ν(C=N) 1578, ν(C–S) 771.

[Cu(L^4^)Cl] (**4**): Green solid. Yield: 87%. Anal. Calc. for C_13_H_21_ClCuN_4_OS (380.40 g mol^−1^): C, 41.05; H, 5.56; Cl, 9.32; Cu, 16.71; N, 14.73; S, 8.43; found: C, 40.96; H, 5.47; Cl, 9.24; Cu, 16.62; N, 14.65; S, 8.33. χ(CH_3_OH): 83 Ω^−1^ cm^−2^ mol^−1^. Main FTIR peaks (cm^−1^): ν(NH) 3364, 3330, ν(C=O) 1613, ν(C=N) 1585, ν(C–S) 774.

[Cu(L^5^)Cl] (**5**): Green solid. Yield: 78%. Anal. Calc. for C_14_H_17_ClCuN_4_O_2_S (404.37 g mol^−1^): C, 41.58; H, 4.24; Cl, 8.77; Cu, 15.71; N, 13.86; S, 7.93; found: C, 41.49; H, 4.16; Cl, 8.68; Cu, 15.61; N, 13.78; S, 7.84. χ(CH_3_OH): 70 Ω^−1^ cm^−2^ mol^−1^. Main FTIR peaks (cm^−1^): ν(NH) 3314, 3332, ν(C=O) 1614, ν(C=N) 1560, ν(C–S) 772.

[Cu(L^6^)Cl] (**6**): Green solid. Yield: 89%. Anal. Calc. for C_11_H_19_ClCuN_4_O_2_S (370.36 g mol^−1^): C, 35.67; H, 5.17; Cl, 9.57; Cu, 17.16; N, 15.13; S, 8.66; found: C, 35.57; H, 5.08; Cl, 9.49; Cu, 17.07; N, 15.04; S, 8.56. χ(CH_3_OH): 88 Ω^−1^ cm^−2^ mol^−1^. Main FTIR peaks (cm^−1^): ν(NH) 3311, 3331, ν(C=O) 1617, ν(C=N) 1581, ν(C–S) 769.

### 3.3. X-Ray Crystallography

Single-crystal X-ray diffraction measurements for **HL^2^**, **HL^4^**, and **HL^6^** were carried out at 293 K using an Xcalibur E diffractometer (Oxford Diffraction Ltd., Abingdon, UK) equipped with a CCD area detector and a graphite monochromator. MoKα radiation (λ = 0.71073 Å) was used as the X-ray source. The CrysAlis PRO CCD software package, version 1.171.37.35 (Oxford Diffraction, Yarnton, Oxfordshire, UK) was employed for data collection, unit cell refinement, and processing of the measured intensities [31].

The crystal structures were determined by direct methods and subsequently refined using the SHELXS97 and SHELXL2014 software packages [32,33]. Refinement was performed by full-matrix least-squares calculations based on F^2^ values. All non-hydrogen atoms were refined with anisotropic displacement parameters. Hydrogen atoms attached to carbon atoms were introduced at geometrically calculated positions and treated using the riding model with standard SHELXL constraints. The isotropic displacement parameters were assigned as Uiso(H) = 1.2 Ueq(C) for CH groups and Uiso(H) = 1.5 Ueq(C) for methyl groups.

The crystallographic parameters and refinement details for **HL^2^**, **HL^4^**, and **HL^6^** are collected in Table S2. Selected geometrical parameters, including bond lengths and angles, are presented in Table S3, while hydrogen-bonding parameters are summarized in Table S1. Molecular graphics were prepared using MERCURY software (version 2025.2.0, Build 454209; Cambridge Crystallographic Data Centre, Cambridge, UK, 2025).

### 3.4. Anticancer Activity Assay

Cell Culture

The human cancer cell lines THP-1 (ATCC: TIB-202), PC-3 (ATCC: CRL-1435), and HT-29 (ATCC: HTB-38), together with the normal epithelial cell line hTERT-RPE1 (ATCC: CRL-4000), were purchased from the American Type Culture Collection (ATCC, Manassas, VA, USA). THP-1, PC-3, and HT-29 cells were grown in RPMI-1640 medium (Sigma, Saint Louis, MO, USA), whereas hTERT-RPE1 cells were maintained in DMEM supplemented with L-glutamine (2 mM), penicillin (100 IU/mL), streptomycin (100 µg/mL), and fetal bovine serum (10%, *v*/*v*).

All cell lines were cultured at 37 °C under a humidified atmosphere containing 5% CO_2_. The growth medium was replaced every 2–3 days during the cultivation period.

MTS Method

To evaluate the half-maximal inhibitory concentration (IC_50_) of the tested compounds on cancer cell lines HT-29, TPH-1, and PC-3 and the normal epithelial cell line hTERT1-RPE1, cells were seeded at a density of 1 × 10^4^ cells per well in 96-well plates. Each well was filled with 90 µL of the appropriate culture medium. Following seeding, the plates were incubated for 24 h at 37 °C in a humidified atmosphere containing 5% CO_2_.

The compounds under investigation were initially dissolved in dimethyl sulfoxide (DMSO) to create stock solutions at a concentration of 1 × 10^−2^ M. The stock solutions were subsequently diluted to a range of concentrations using culture medium. A volume of 10 µL of each dilution was added to the wells, and the cells were incubated with the compounds for 48 h.

Following treatment, 20 µL of 3-(4,5-dimethylthiazol-2-yl)-5-(3-carboxymethoxyphenyl)-2-(4-sulfophenyl)-2*H*-tetrazolium (MTS) (the final concentration of MTS was 0.33 mg/mL) was added to each well, and the mixture was incubated for 4 h. Absorbance was measured at 490 nm using a microplate reader (Molecular Devices, Sunnyvale, CA, USA). The IC_50_ values were calculated using GraphPad Prism 6 software.

### 3.5. Antibacterial and Antifungal Activity Assays

The antibacterial and antifungal properties of ligands **HL^1^**^–**5**^ and their corresponding copper(II) coordination compounds **1**–**5** were evaluated using reference microbial strains, namely *Staphylococcus aureus* (ATCC 25923), *Bacillus cereus* (ATCC 11778), and *Candida albicans* (ATCC 10231). Antimicrobial efficacy was assessed by the liquid broth microdilution technique, which enabled the determination of minimum inhibitory (MIC), minimum bactericidal (MBC), and minimum fungicidal (MFC) concentrations, expressed in μg mL^−1^. The investigated compounds were initially dissolved in DMSO to obtain stock solutions with a concentration of 10 mg mL^−1^, followed by serial dilutions prepared in 2% peptone broth. Tetracycline [34,35,36,37] and fluconazole [38] were employed as reference drugs for antibacterial and antifungal activity, respectively.

### 3.6. Antioxidant Activity Assay

Antioxidant properties were evaluated using the ABTS^•+^ radical cation decolorization assay, a widely applied method suitable for assessing both hydrophilic and lipophilic antioxidants [39]. The preparation of the ABTS^•+^ radical solution, experimental conditions, spectrophotometric measurements, and inhibition calculations were carried out according to established protocols. Stock solutions (10 mM) of the investigated compounds as well as Trolox, used as a reference antioxidant, were prepared by dissolving 10 μmol of each substance in 1 mL of DMSO. Working solutions with concentrations of 1, 10, 100, and 1000 μM were obtained by serial dilution with DMSO. Subsequently, 20 μL of each sample solution was dispensed into the wells of a 96-well microplate, followed by the addition of 180 μL of the ABTS^•+^ working solution, yielding final test concentrations of 0.1, 1, 10, and 100 μM.

## 4. Conclusions

In this work, the synthesis and characterization of six 4-allylthiosemicarbazones are reported. The obtained results demonstrate that copper(II) coordination has a distinct and activity-dependent effect on the biological properties of the studied thiosemicarbazones. In particular, metal coordination generally enhances antiproliferative and antimicrobial activities, whereas it decreases the ABTS^•+^ radical-scavenging activity. No direct correlation between antioxidant, antimicrobial, and antiproliferative activities was observed, indicating that these biological effects are likely governed by different molecular and cellular mechanisms. The observed differences further emphasize the important role of the substituent in the pyruvamide fragment in modulating the biological profile of both the free ligands and their copper(II) complexes. These findings provide a basis for further structural optimization aimed not only at increasing biological potency but also at improving selectivity toward cancer cells and achieving a more favorable overall biological profile.

## Figures and Tables

**Table 1:** Antiproliferative activities of the synthesized compounds against the human leukemia THP-1 cell line at three concentrations.

Compound	Inhibition of Cell Proliferation (%)			IC_50_, µM
	10 µM	1 µM	0.1 µM	
**HL^1^**	39.9 ± 0.6	24.0 ± 0.4	3.9 ± 0.9	>10
**1**	87.8 ± 0.1	39.9 ± 0.2	29.7 ± 0.9	0.95 ± 0.02
**HL^2^**	53.8 ± 1.3	48.5 ± 0.1	10.5 ± 0.1	3.85 ± 0.36
**2**	79.8 ± 1.0	49.3 ± 1.2	33.8 ± 1.0	0.66 ± 0.08
**HL^3^**	53.7 ± 1.0	51.0 ± 0.1	28.8 ± 0.2	2.79 ± 0.28
**3**	88.6 ± 0.2	62.0 ± 0.4	49.6 ± 0.9	0.14 ± 0.01
**HL^4^**	29.9 ± 1.6	28.7 ± 3.0	26.2 ± 7.9	>10
**4**	37.2 ± 3.7	16.8 ± 1.3	9.0 ± 2.5	>10
**HL^5^**	24.7 ± 1.0	3.7 ± 2.1	2.2 ± 0.2	>10
**5**	53.0 ± 0.6	35.9 ± 0.5	42.3 ± 2.4	5.64 ± 1.50
**HL^6^**·0.5H_2_O	37.2 ± 1.9	31.1 ± 2.3	9.6 ± 0.1	>10
**6**	71.9 ± 1.0	43.1 ± 1.6	42.7 ± 0.1	0.69 ± 0.04
Doxorubicin	98.3 ± 0.4	75.0 ± 6.7	24.7 ± 0.8	0.32 ± 0.06

**Table 2:** Antiproliferative activities of the synthesized compounds against the human prostate cancer PC-3 cell line at three concentrations.

Compound	Inhibition of Cell Proliferation (%)			IC_50_, µM
	10 µM	1 µM	0.1 µM	
**HL^1^**	20.1 ± 0.8	19.5 ± 2.0	25.1 ± 1.2	>10
**1**	101.5 ± 0.4	24.3 ± 3.4	25.8 ± 1.5	1.58 ± 0.06
**HL^2^**	67.7 ± 5.6	36.6 ± 9.2	33.9 ± 5.2	2.17 ± 1.28
**2**	95.9 ± 3.4	28.3 ± 3.0	24.8 ± 2.0	1.61 ± 0.22
**HL^3^**	4.6 ± 1.5	4.7 ± 3.4	4.8 ± 2.7	>10
**3**	98.4 ± 0.7	5.0 ± 6.1	3.7 ± 1.7	2.95 ± 1.08
**HL^4^**	32.6 ± 0.1	31.9 ± 7.3	32.7 ± 4.6	>10
**4**	34.8 ± 7.7	27.5 ± 2.9	9.2 ± 5.2	>10
**HL^5^**	8.9 ± 4.2	7.8 ± 0.7	4.8 ± 4.0	>10
**5**	61.0 ± 0.5	7.7 ± 5.0	5.4 ± 2.3	7.02 ± 0.68
**HL^6^**·0.5H_2_O	22.0 ± 1.9	26.6 ± 2.2	26.3 ± 2.9	>10
**6**	32.4 ± 1.5	23.3 ± 1.2	11.1 ± 6.5	>10
Doxorubicin	79.5 ± 7.5	70.6 ± 1.1	48.6 ± 5.5	0.828 ± 0.66

**Table 3:** Antiproliferative activities of the synthesized compounds against the human colorectal cancer HT-29 cell line at three concentrations.

Compound	Inhibition of Cell Proliferation (%)			IC_50_, µM
	10 µM	1 µM	0.1 µM	
**HL^1^**	2.5 ± 2.8	3.2 ± 1.9	3.6 ± 0.7	>10
**1**	62.9 ± 2.9	4.3 ± 2.8	1.8 ± 0.9	7.20 ± 0.02
**HL^2^**	10.7 ± 0.3	16.5 ± 3.6	9.2 ± 0.1	>10
**2**	36.6 ± 5.0	13.4 ± 2.6	21.3 ± 2.6	>10
**HL^3^**	3.9 ± 2.9	4.8 ± 1.2	1.7 ± 1.3	>10
**3**	0.9 ± 0.1	1.7 ± 6.8	4.9 ± 7.8	>10
**HL^4^**	12.7 ± 0.9	14.6 ± 0.7	8.3 ± 3.0	>10
**4**	33.4 ± 2.0	31.9 ± 0.6	13.2 ± 0.7	>10
**HL^5^**	5.4 ± 0.4	2.6 ± 1.5	16.2 ± 1.5	>10
**5**	27.8 ± 2.0	27.7 ± 2.1	15.0 ± 2.5	>10
**HL^6^**·0.5H_2_O	13.1 ± 2.8	13.3 ± 3.4	20.6 ± 6.9	>10
**6**	16.3 ± 1.0	13.3 ± 4.0	12.9 ± 1.0	>10
Doxorubicin	62.6 ± 3.7	46.2 ± 8.5	14.6 ± 5.7	23.65 ± 5.1

**Table 4:** Antibacterial and antifungal activities of **HL^1^**^–**5**^ and copper(II) complexes **1**–**5** as MIC/MBC/MFC values in μg mL^−1^.

Compound	*Staphylococcus aureus* ATCC 25923		*Bacillus cereus* ATCC 11778		*Candida albicans* ATCC 10231	
	MIC	MBC	MIC	MBC	MIC	MFC
**HL^1^**	125.0	250.0	>1000	>1000	500.0	500.0
**1**	31.25	62.50	15.63	31.25	125.0	250.0
**HL^2^**	500.0	500.0	500.0	>1000	250.0	>1000
**2**	62.50	62.50	31.25	62.50	250.0	500.0
**HL^3^** [26]	125.0	250.0	125.0	125.0	125.0	250.0
**3** [26]	125.0	125.0	>1000	>1000	250.0	500.0
**HL^4^**	>1000	>1000	>1000	>1000	>1000	>1000
**4**	31.25	31.25	15.63	15.63	125.0	250.0
**HL^5^**	>1000	>1000	125.0	250.0	>1000	>1000
**5**	15.63	31.25	7.813	15.63	>1000	>1000
Tetracycline	0.25	1.96	0.06	-	-	-
Fluconazole	-	-	-	-	15.6	31.3

**Table 5:** Antioxidant activity of **HL^1^**^–**6**^ and compounds **1**–**6** against cation radicals ABTS^•+^.

Compound	IC_50_, μM
**HL^1^** [25]	56.4 ± 1.5
**1** [25]	105.0 ± 4.5
**HL^2^**	3.85 ± 0.18
**2**	8.16 ± 0.15
**HL^3^** [26]	94.4 ± 4.9
**3** [26]	128.4 ± 3.5
**HL^4^**	84.2 ± 4.5
**4**	≥100
**HL^5^**	68.6 ± 4.6
**5**	90.5 ± 4.2
**HL^6^**·0.5H_2_O	7.71 ± 0.27
**6**	14.2 ± 0.2
Trolox	33.0 ± 1.0

## Data Availability

Data are contained within the article and Supplementary Materials.

## Supplementary Materials

The following supporting information can be downloaded at https://www.mdpi.com/article/10.3390/molecules31162857/s1, Figures S1–S12: ^1^H and ^13^C NMR spectra of **HL^1^**^–**6**^; Figures S13–S24: FTIR spectra of **HL^1^**^–**6**^ and complexes **1**–**6**; Table S1: Hydrogen bond distances (Å) and angles (deg) for **HL^2^, HL^4^** and **HL^6^·0.5H_2_O**; Table S2: Crystal and structure refinement data for **HL^2^**, **HL^4^**, and **HL^6^·0.5H_2_O**; Table S3: Selected bond lengths (Å) and angles (deg) in **HL^2^**, **HL^4^**, and **HL^6^·0.5H_2_O**; Figure S25: Molecular structure of compound **HL^2^** with notation of the common fragment to three structures.
