# Supplementary material for: Synthesis and Biological Evaluation of a Series of N-Substituted Pyruvamide 4-Allylthiosemicarbazones and Their Copper(II) Complexes: Antibacterial, Antiradical, and Antiproliferative Activities
Source: Molecules. 2026 Aug 16;31(16):2857. doi: 10.3390/molecules31162857 (PMC13515737; doi:10.3390/molecules31162857)
Supplement: Supplementary file 1 [file molecules-31-02857-s001.zip › molecules-4439250-supplementary.pdf]

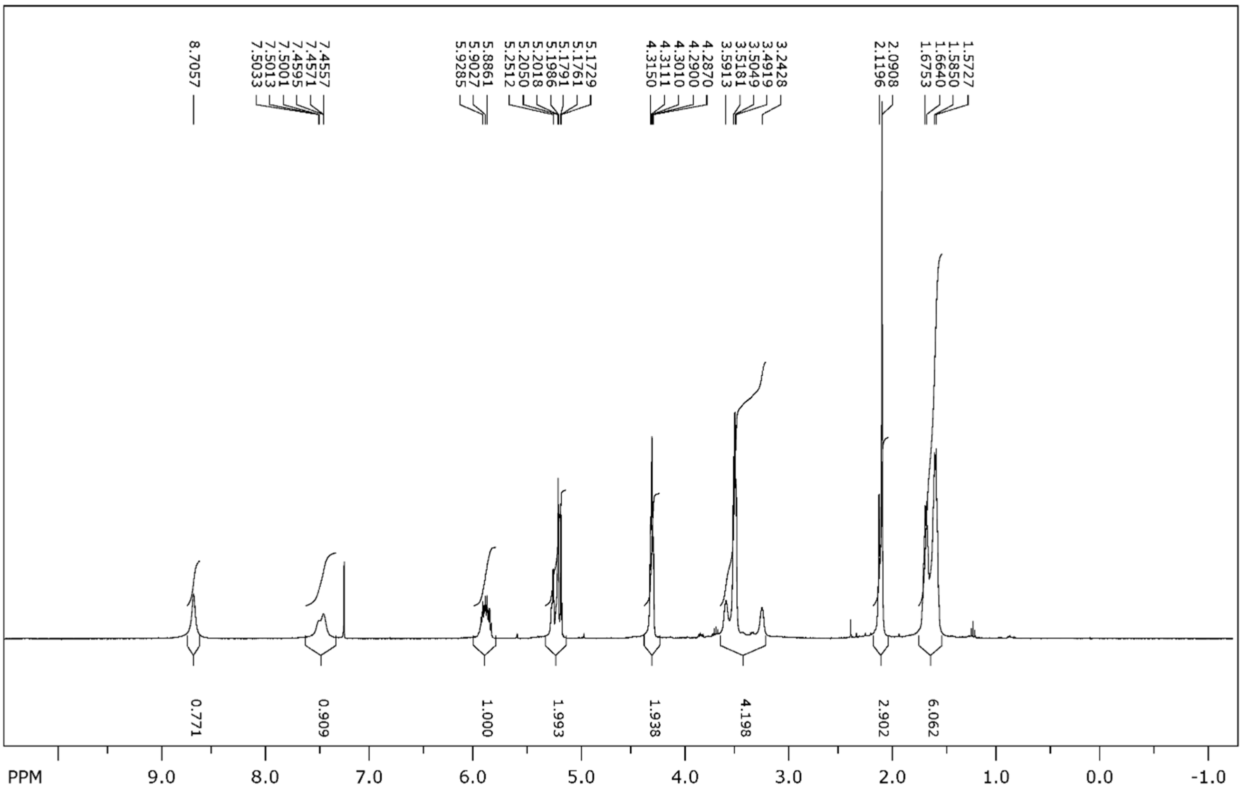

Figure S1. ^{1}H NMR (400 MHz, $CDCl_3$) spectrum of 3-(piperidin-1-yl)propane-2,3-dione 4-allylthiosemicarbazone (**HL^1**).

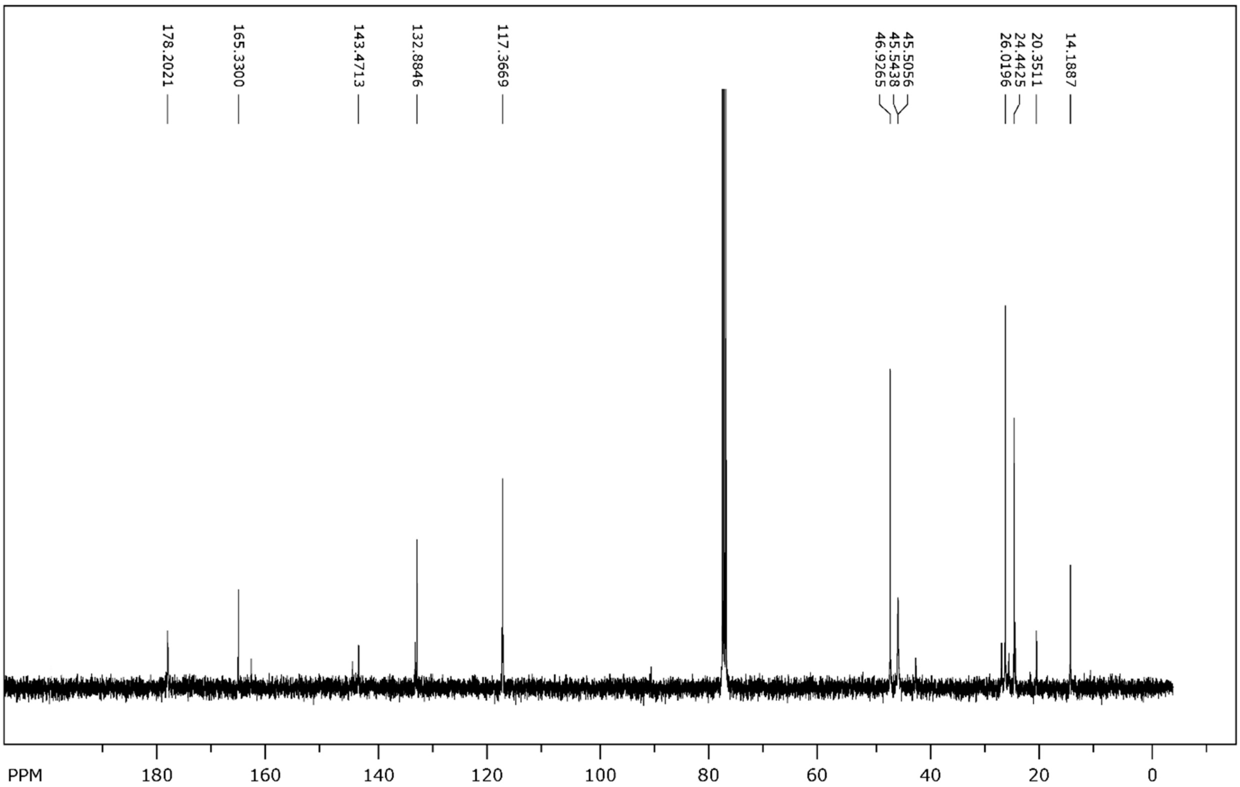

Figure S2. ^{13}C NMR (100 MHz, $CDCl_3$) spectrum of 3-(piperidin-1-yl)propane-2,3-dione 4-allylthiosemicarbazone (**HL^1**).

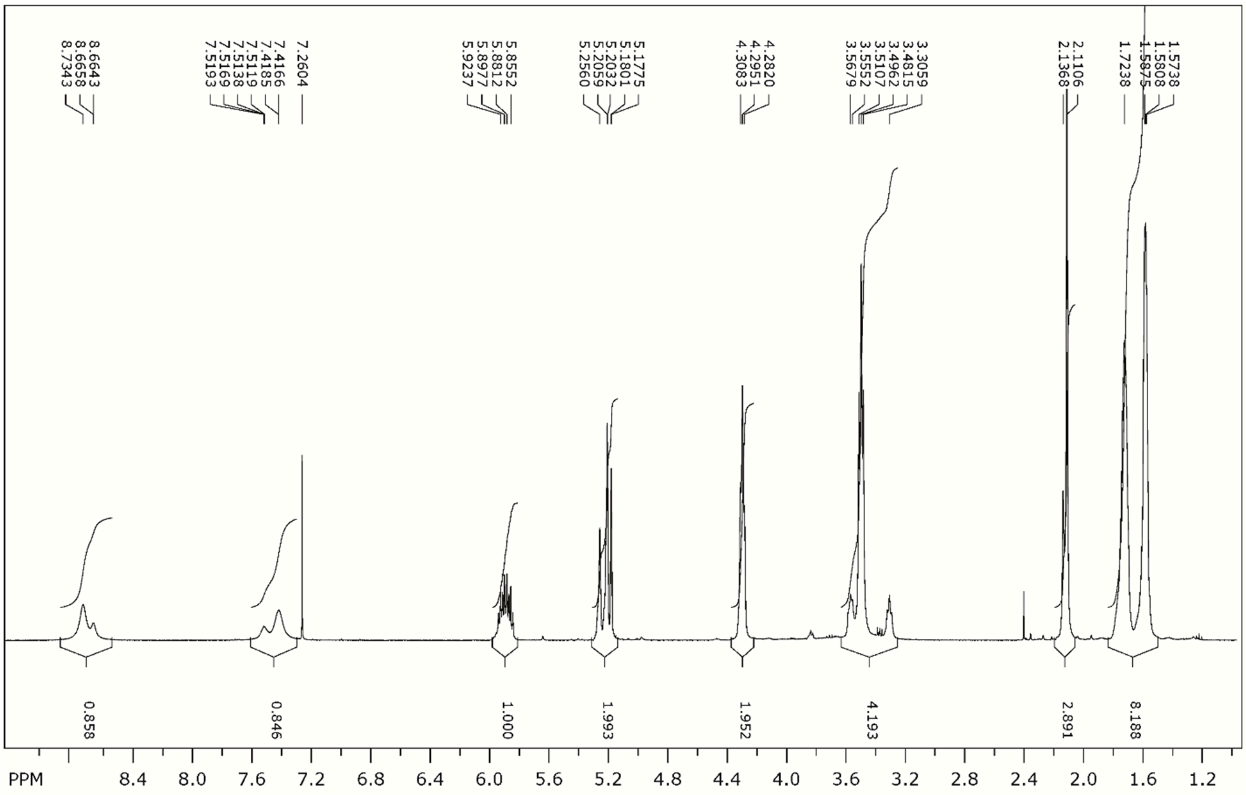

Figure S3. ^{1}H NMR (400 MHz, $CDCl_3$) spectrum of 3-(azepan-1-yl)propane-2,3-dione 4-allylthiosemicarbazone (**HL^2**).

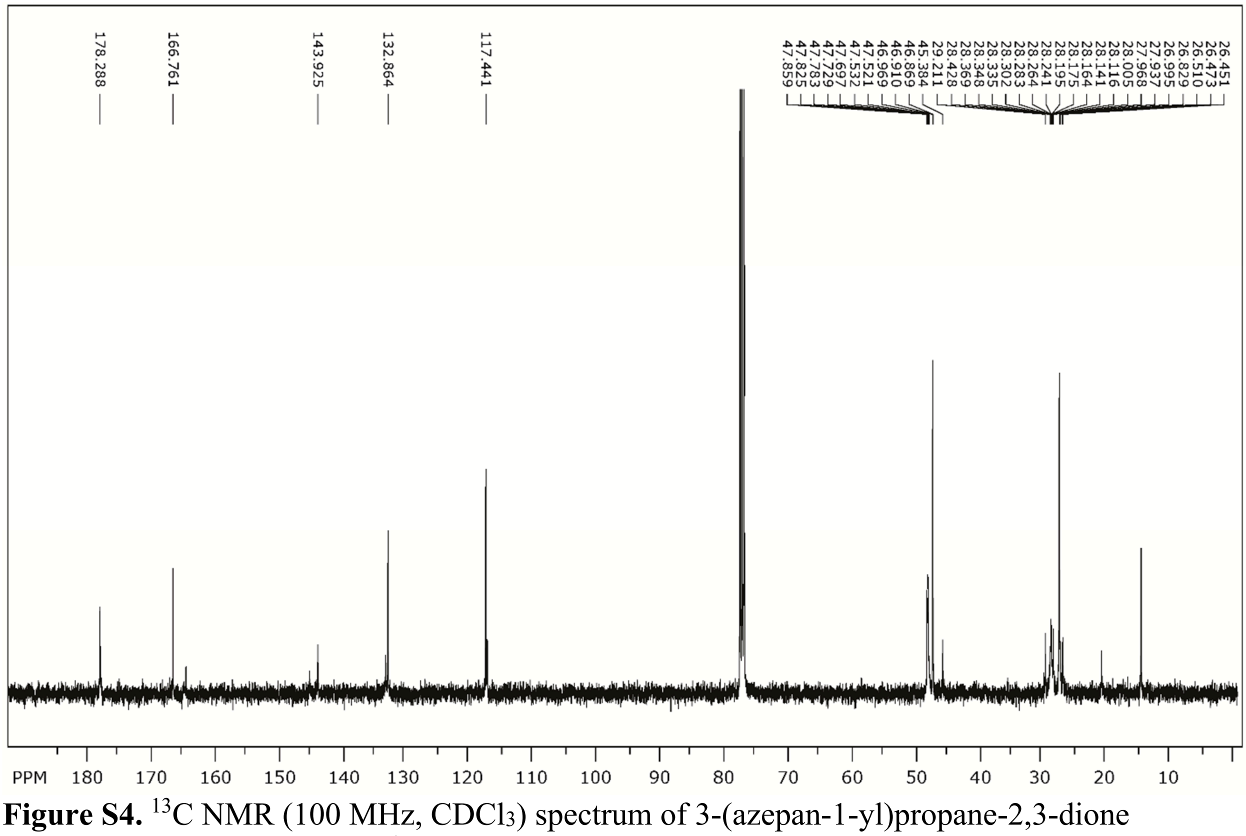

Figure S4. ^{13}C NMR (100 MHz, $CDCl_3$) spectrum of 3-(azepan-1-yl)propane-2,3-dione 4-allylthiosemicarbazone (**HL^2**).

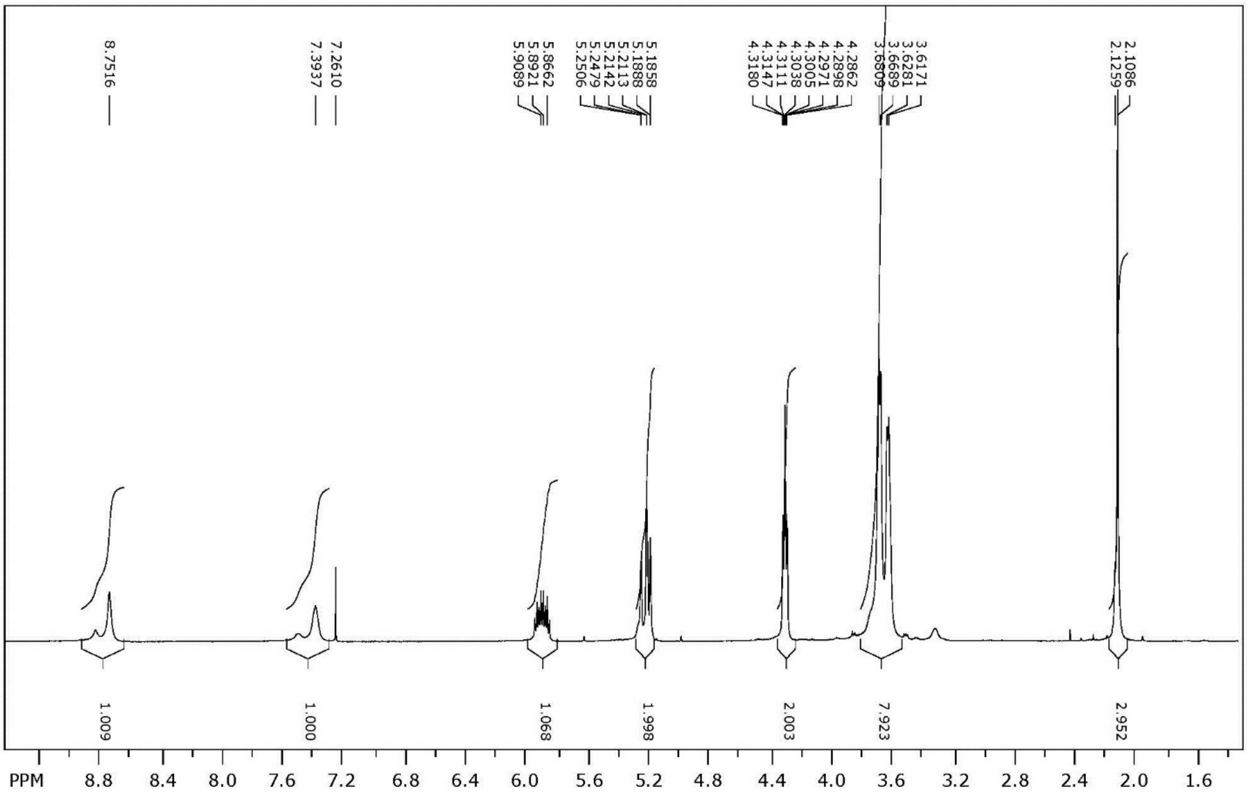

Figure S5. ^{1}H NMR (400 MHz, $CDCl_3$) spectrum of 3-(morpholin-4-yl)propane-2,3-dione 4-allylthiosemicarbazone (**HL^{3}**).

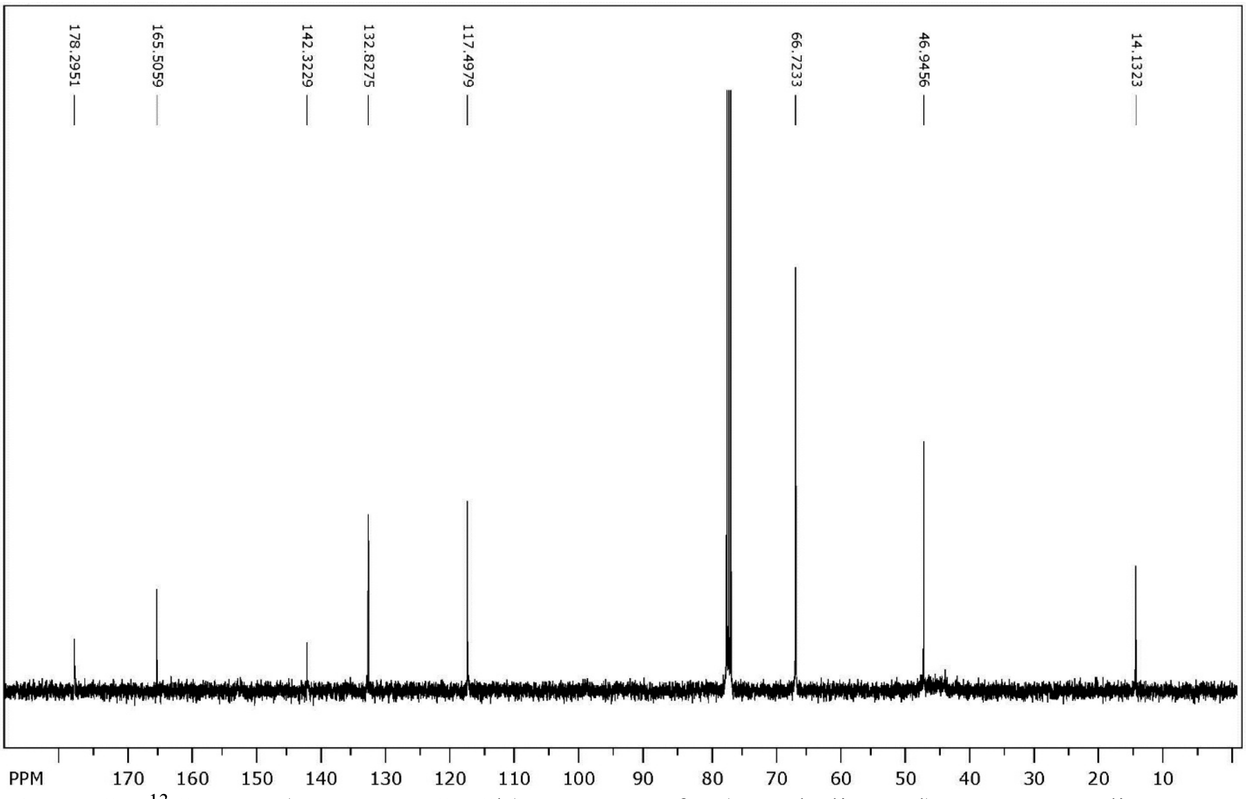

Figure S6. ^{13}C NMR (100 MHz, $CDCl_3$) spectrum of 3-(morpholin-4-yl)propane-2,3-dione 4-allylthiosemicarbazone (**HL^{3}**).

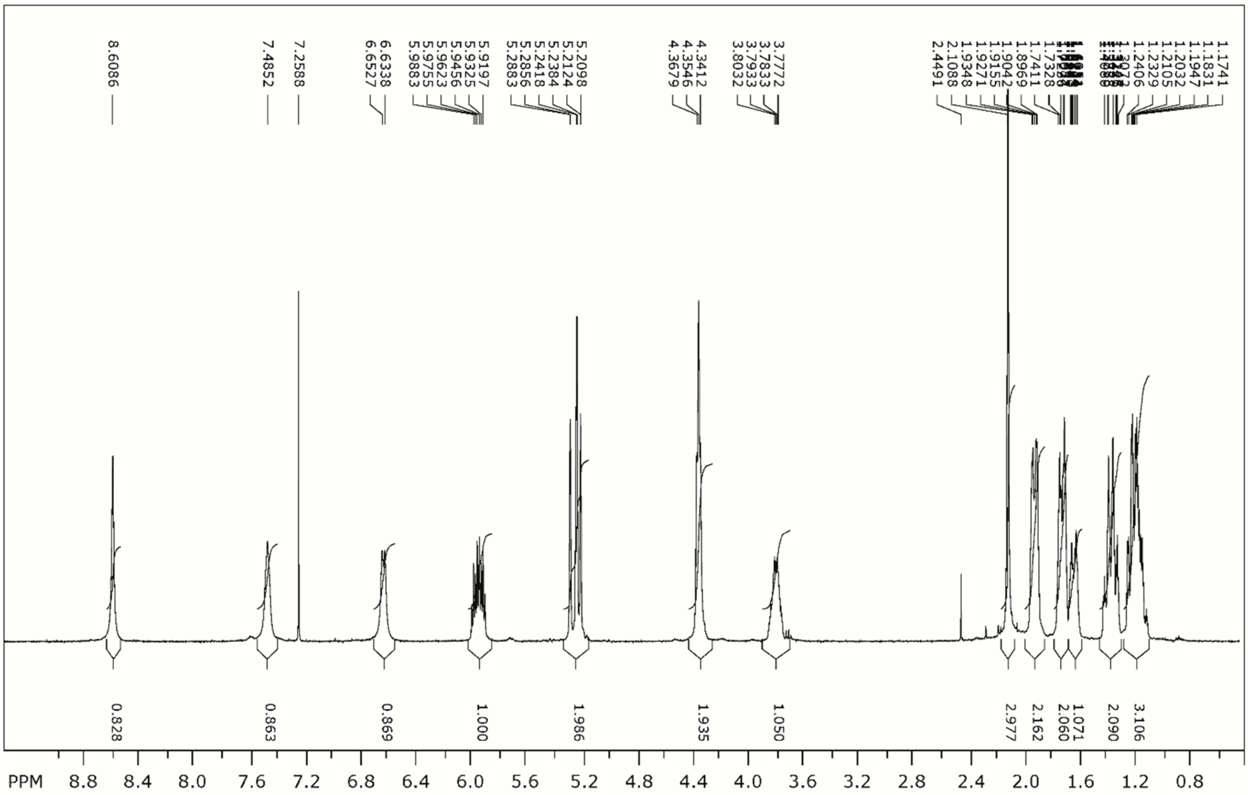

Figure S7. ^{1}H NMR (400 MHz, $CDCl_3$) spectrum of *N*-cyclohexyl-2-oxopropanamide 4-allylthiosemicarbazone (**HL^4**).

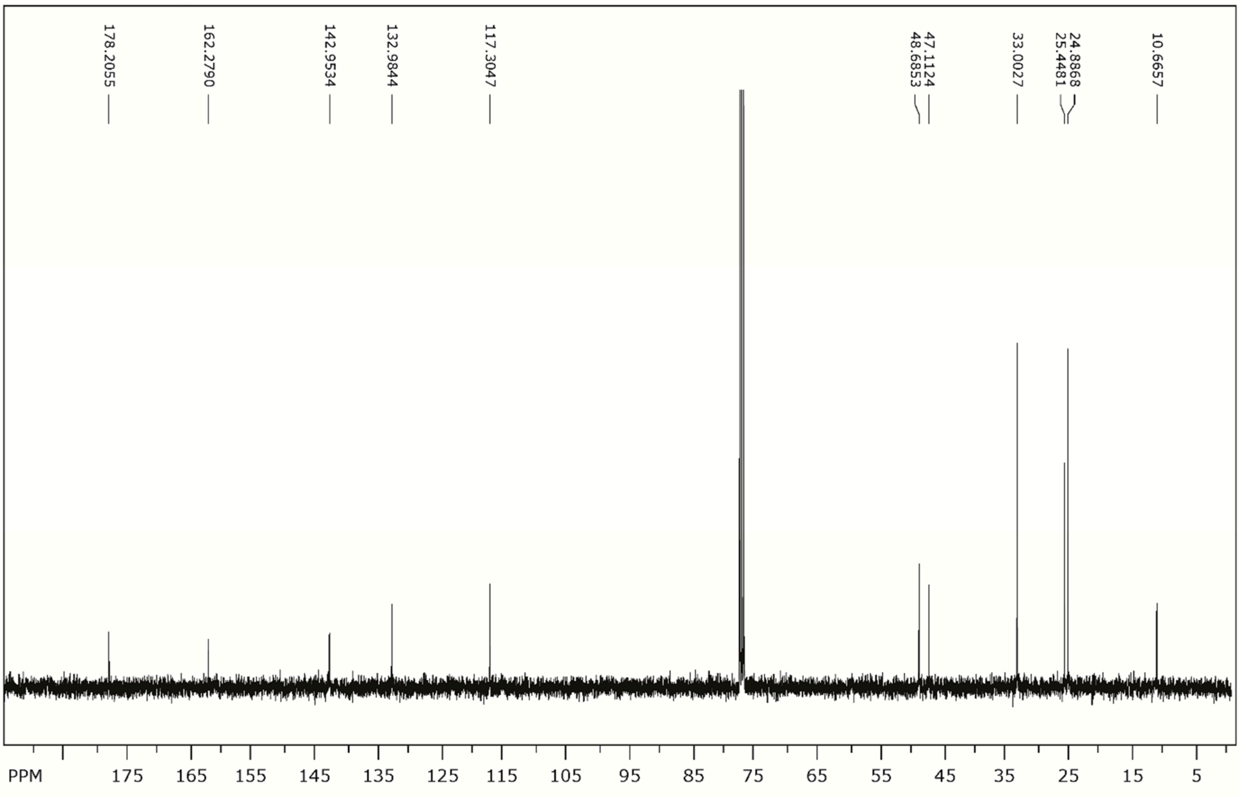

Figure S8. ^{13}C NMR (100 MHz, $CDCl_3$) spectrum of *N*-cyclohexyl-2-oxopropanamide 4-allylthiosemicarbazone (**HL^4**).

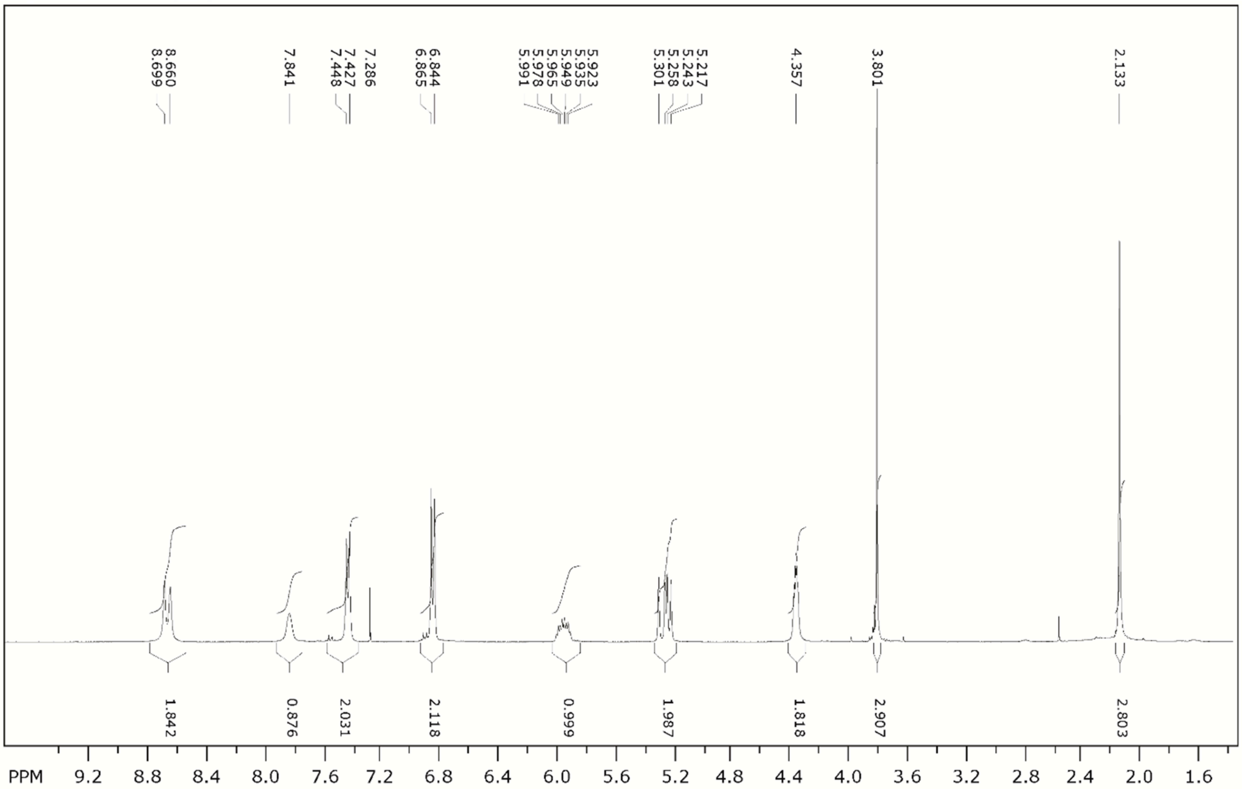

Figure S9. ^{1}H NMR (400 MHz, $CDCl_3$) spectrum of *N*-(4-methoxyphenyl)-2-oxopropanamide 4-allylthiosemicarbazone (**HL**^{5}).

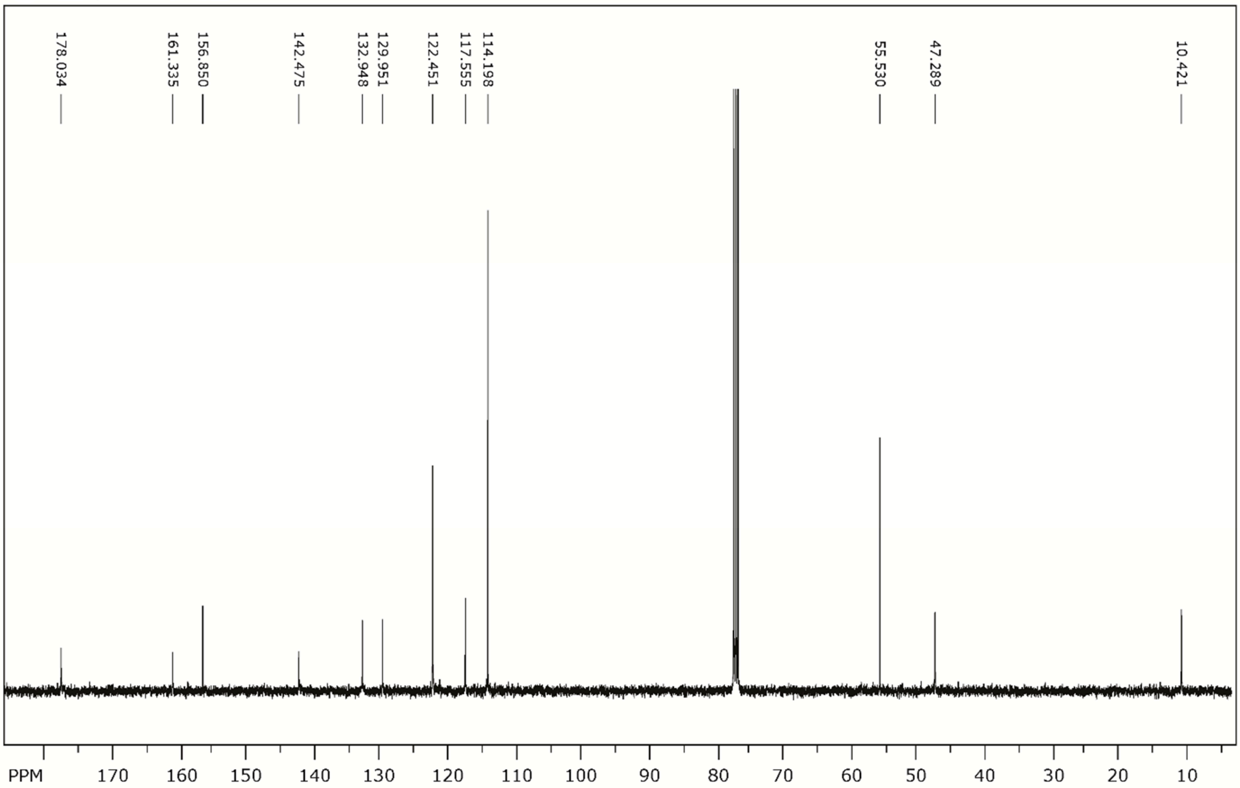

Figure S10. ^{13}C NMR (100 MHz, $CDCl_3$) spectrum of *N*-(4-methoxyphenyl)-2-oxopropanamide 4-allylthiosemicarbazone (**HL**^{5}).

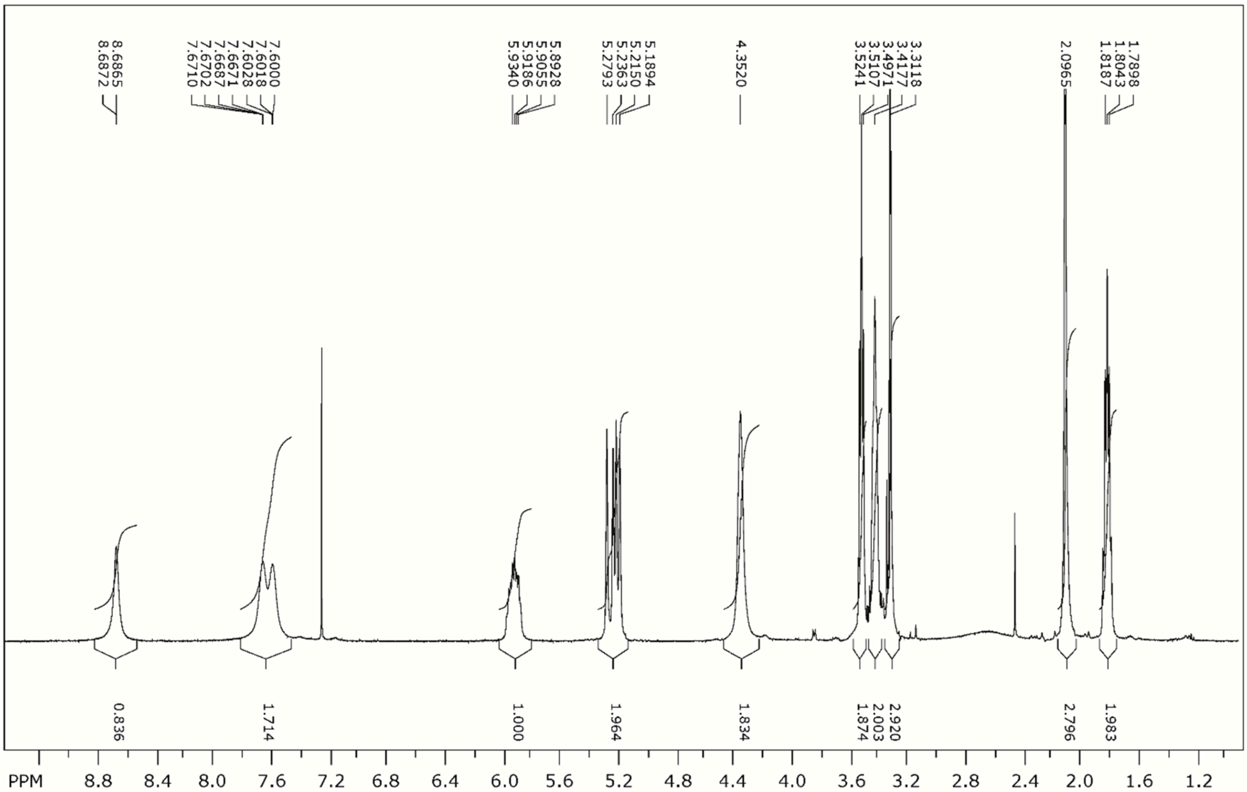

Figure S11. ^{1}H NMR (400 MHz, $CDCl_3$) spectrum of *N*-(4-methoxyphenyl)-2-oxopropanamide 4-allylthiosemicarbazone (**HL^{6}**).

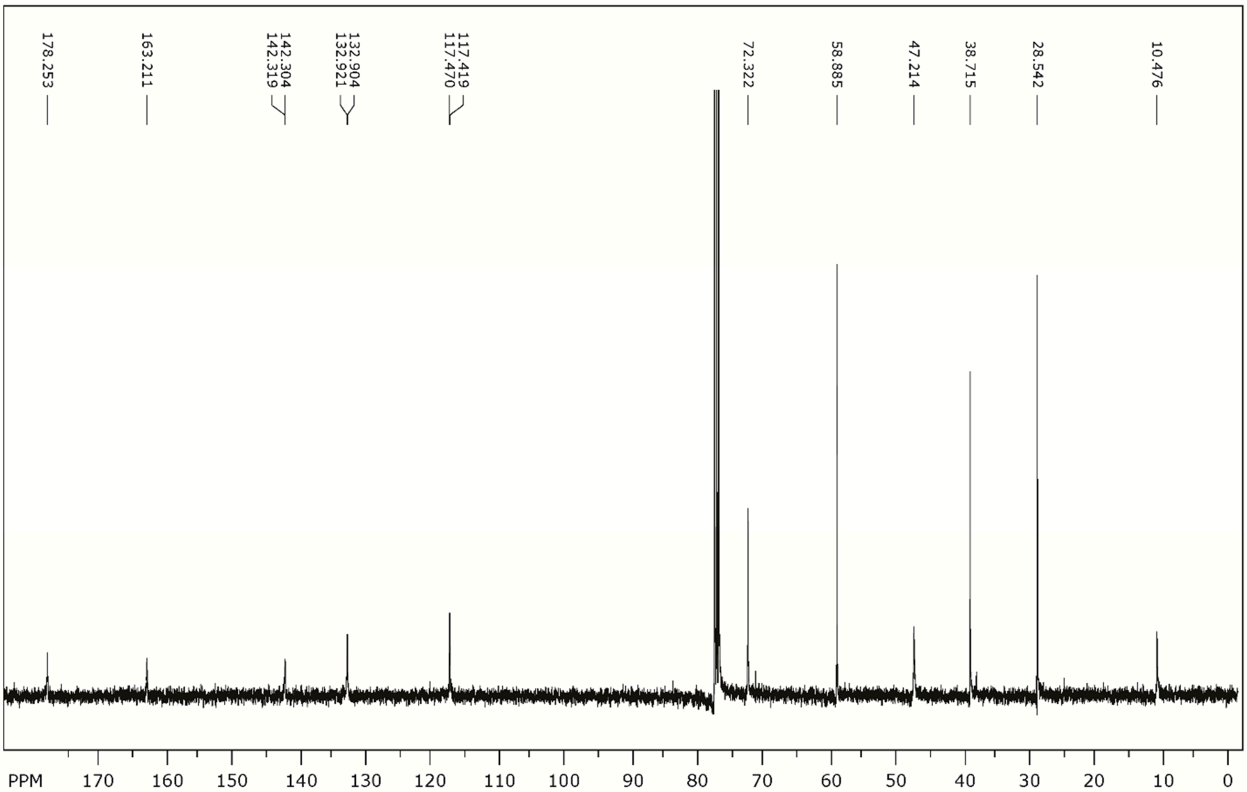

Figure S12. ^{13}C NMR (100 MHz, $CDCl_3$) spectrum of *N*-(4-methoxyphenyl)-2-oxopropanamide 4-allylthiosemicarbazone (**HL^{6}**).

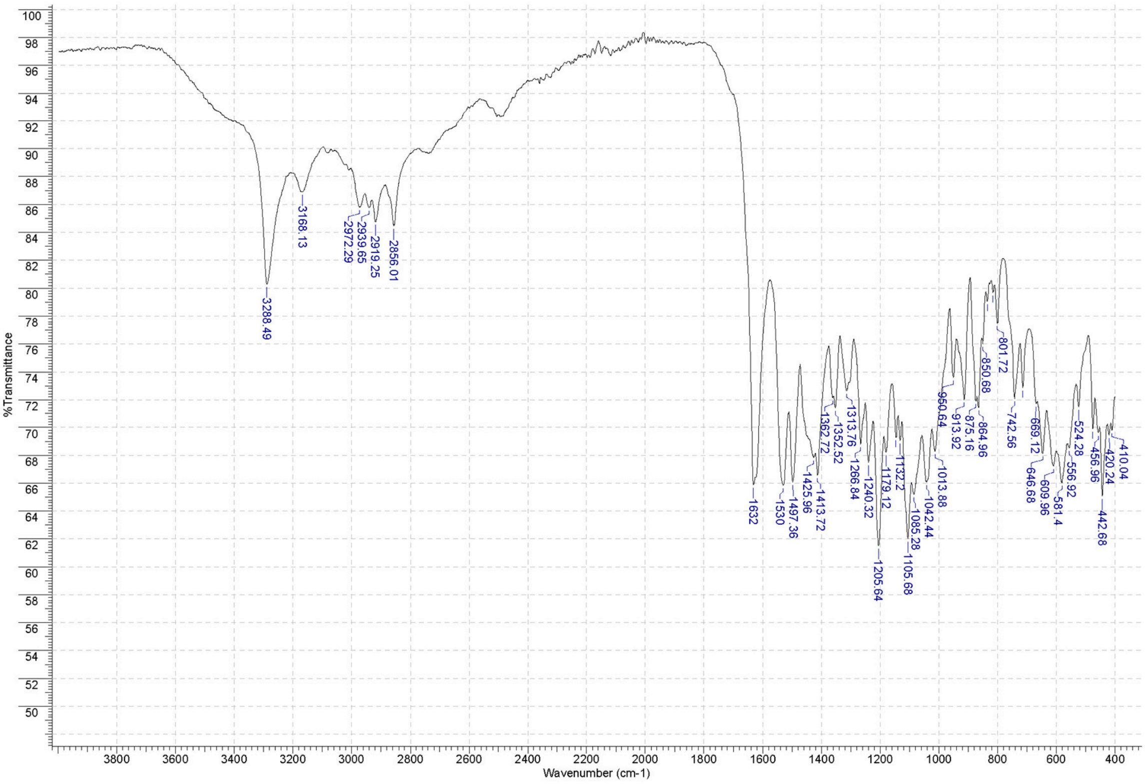

Figure S13. FTIR spectrum of **HL**[1].

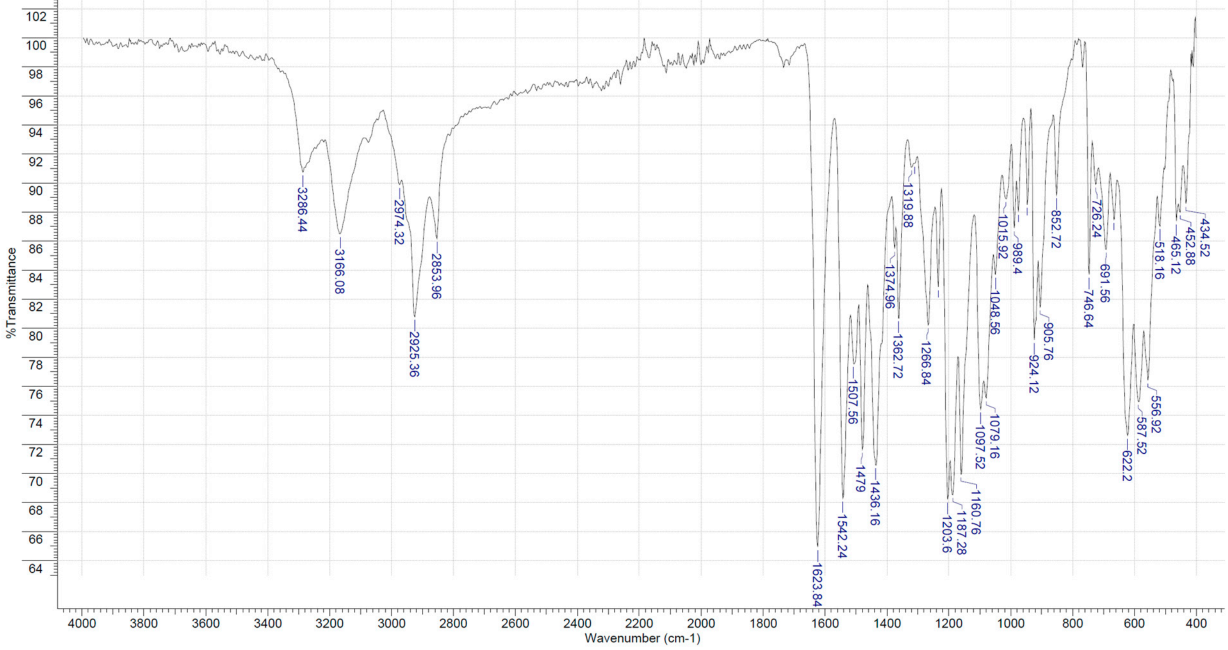

Figure S14. FTIR spectrum of **HL**[2].

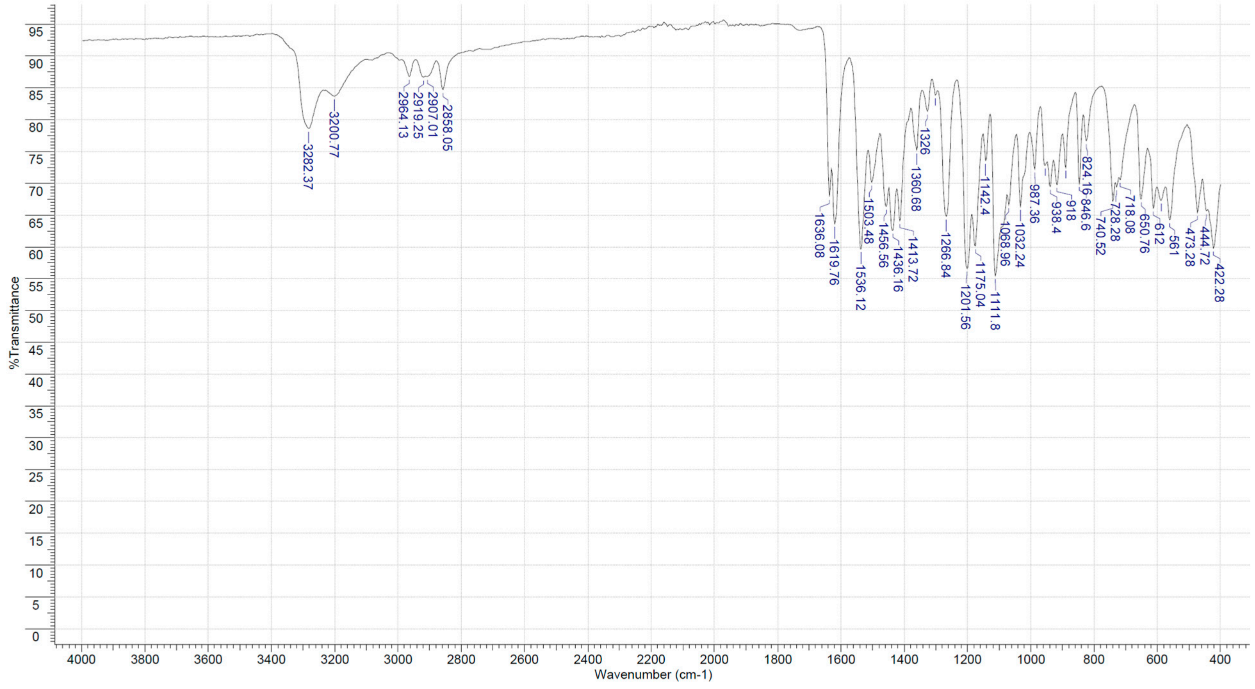

Figure S15. FTIR spectrum of **HL**^{3}.

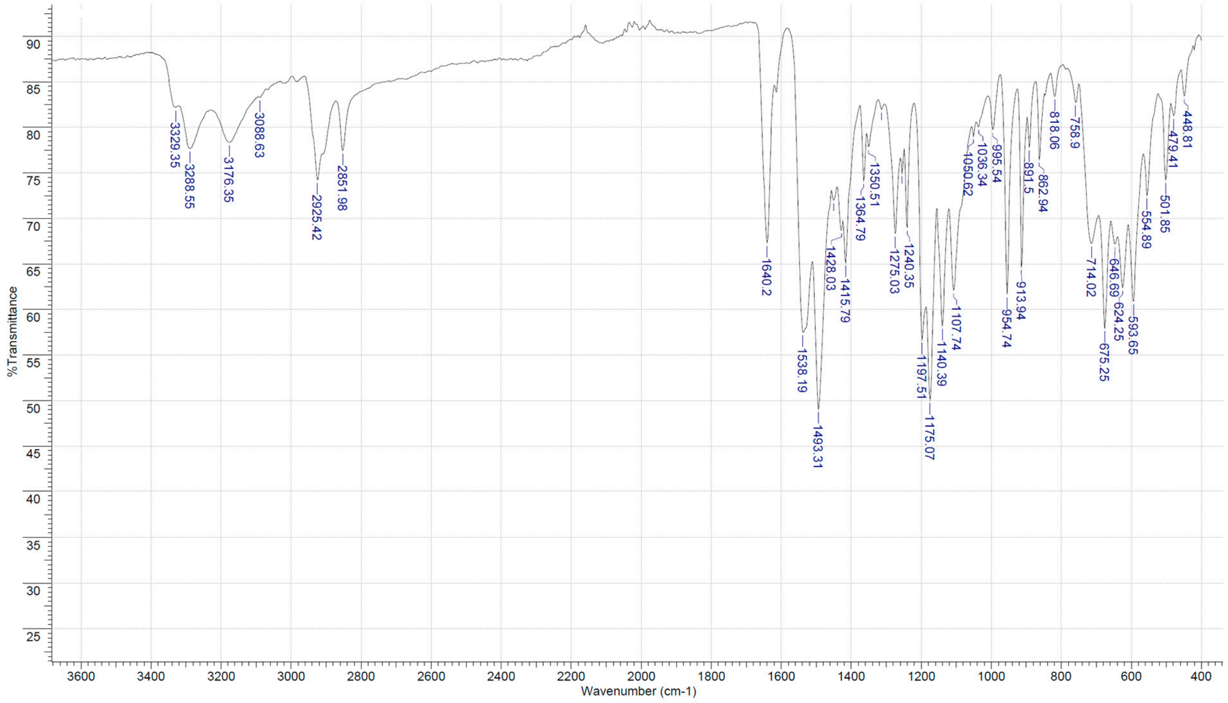

Figure S16. FTIR spectrum of **HL**^{4}.

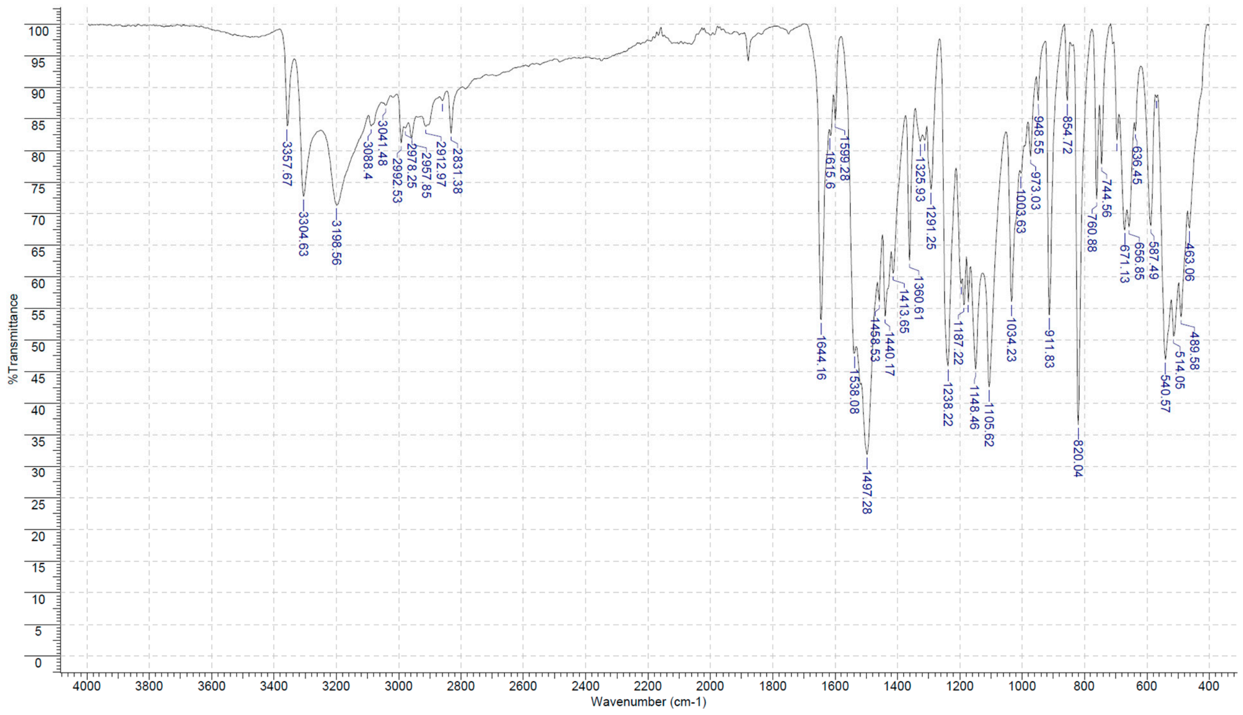

Figure S17. FTIR spectrum of **HL**[5].

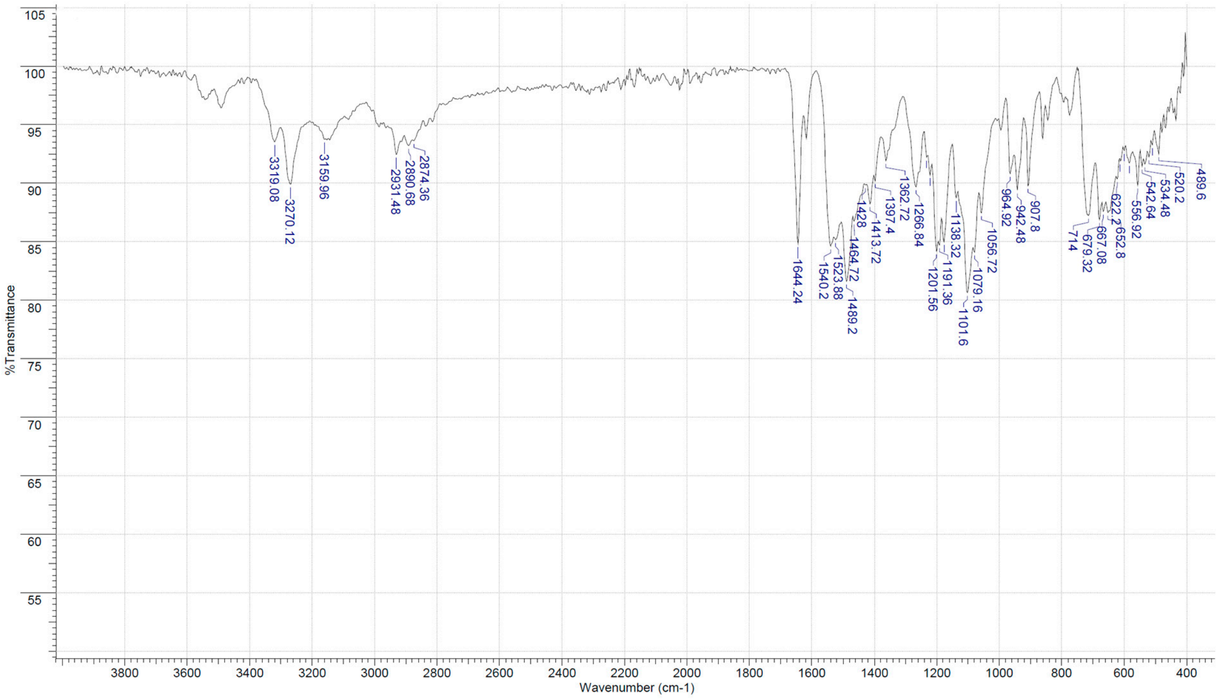

Figure S18. FTIR spectrum of **HL**[6].

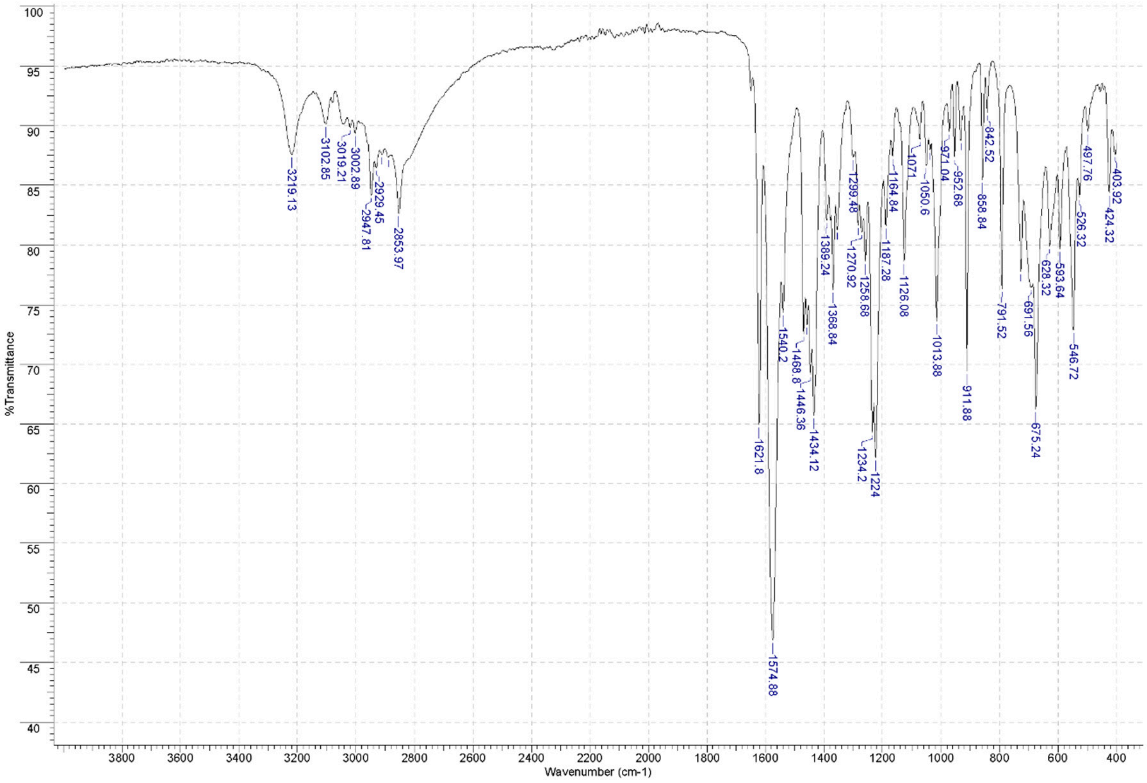

Figure S19. FTIR spectrum of complex **1**.

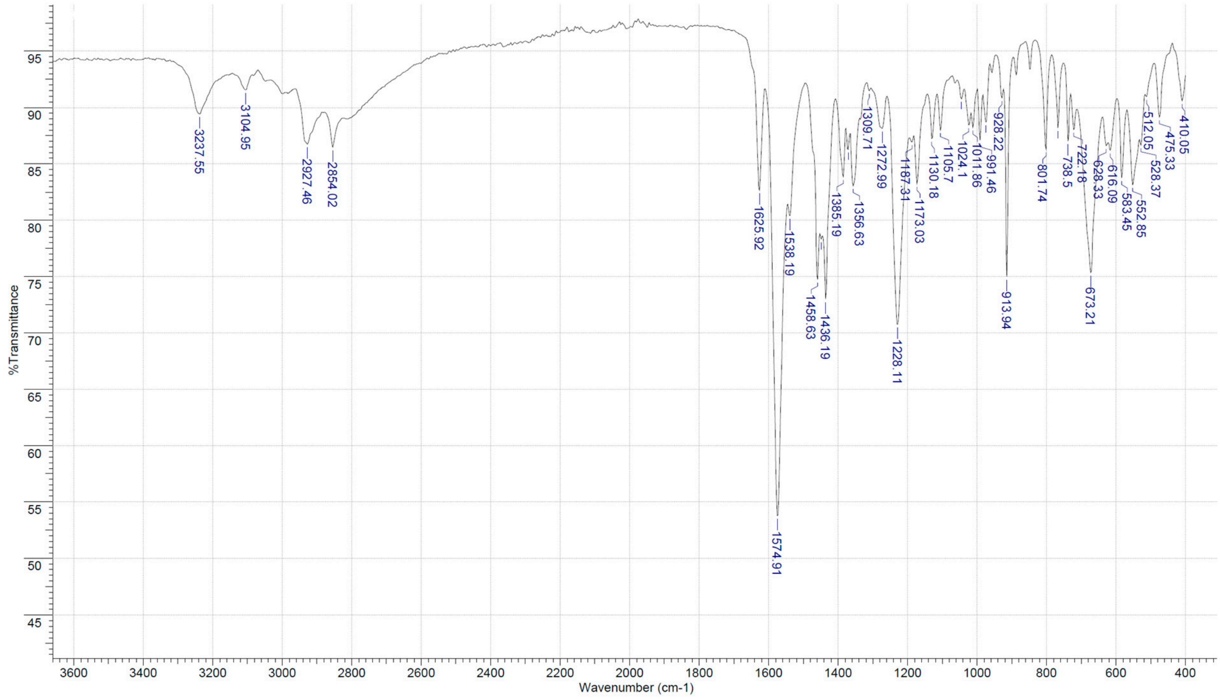

Figure S20. FTIR spectrum of complex **2**.

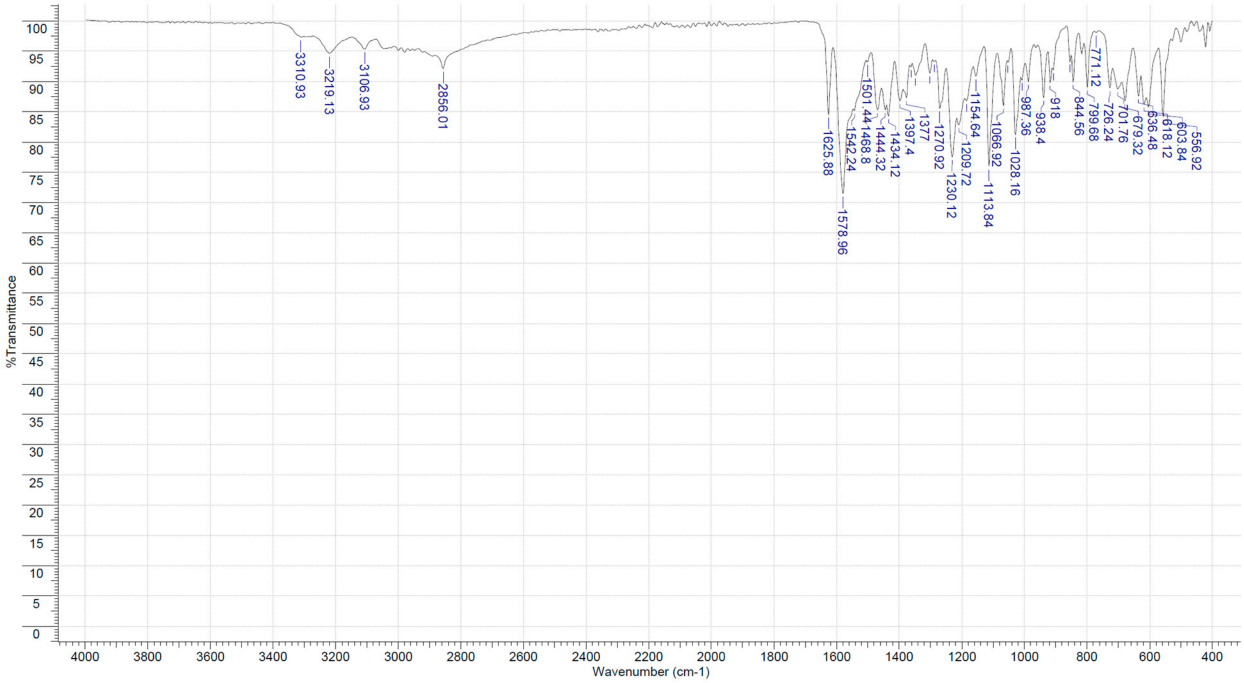

Figure S21. FTIR spectrum of complex **3**.

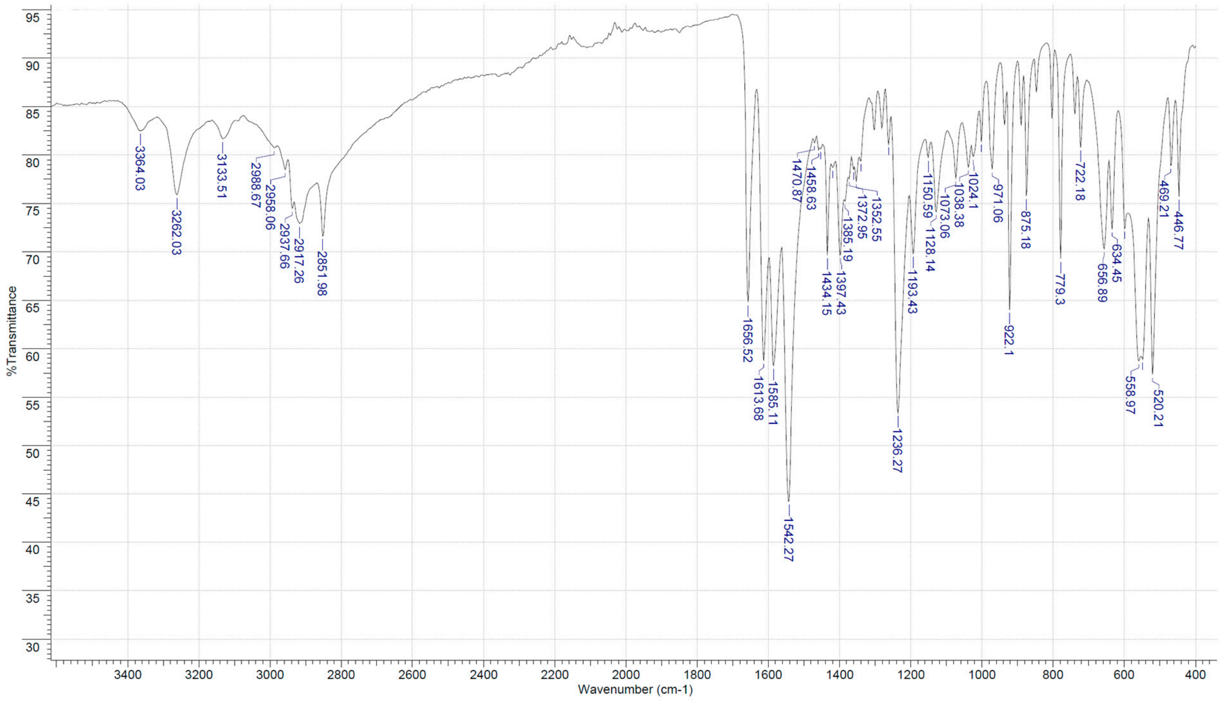

Figure S22. FTIR spectrum of complex **4**.

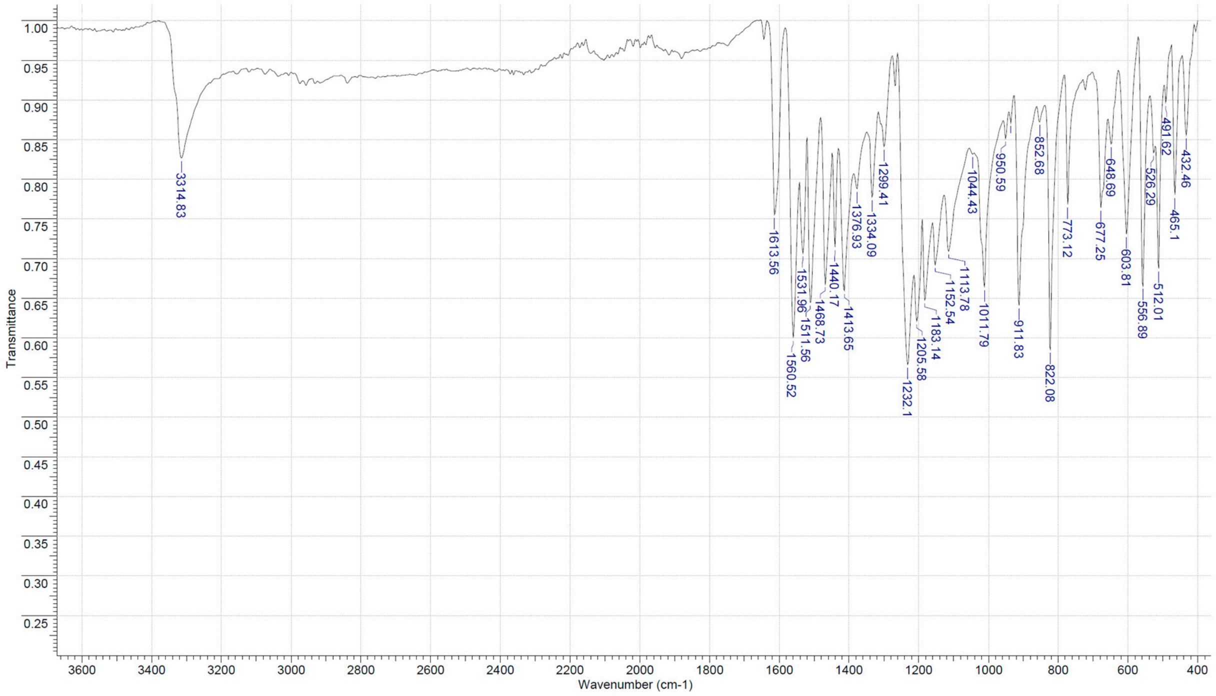

Figure S23. FTIR spectrum of complex **5**.

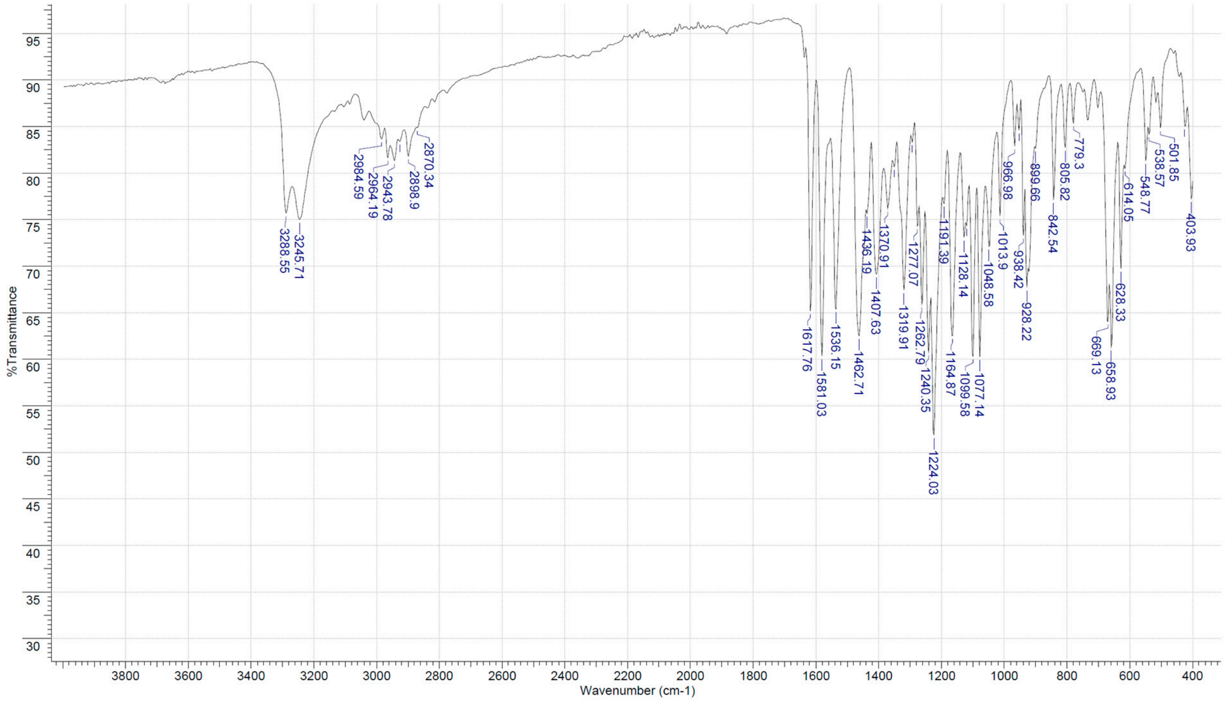

Figure S24. FTIR spectrum of complex **6**.

Table S1. Hydrogen Bond Distances (Å) and Angles (deg) for $\mathbf{HL^2}$, $\mathbf{HL^4}$ and $\mathbf{HL^6 \cdot 0.5H_2O}$.

D–H···A	d(H···A)	d(D···A)	∠(DHA)	Symmetry transformation for acceptor
**$\mathbf{HL^2}$**				
N(4)–H(4)···N(2)	2.17	2.582(3)	109	*x*, *y*, z
N(3)–H(3)···S(1)	2.65	3.498(2)	168	–*x*+1, –*y*+2, –*z*+1
N(4)–H(4)···O(1)	2.27	2.938(3)	135	–*x*+1, –*y*+1, –*z*
**$\mathbf{HL^4}$**				
N(4)–H(4)···N(2)	2.19	2.581(5)	108	*x*, *y*, z
N(1)–H(4)···N(2)	2.23	2.622(4)	108	*x*, *y*, z
N(1)–H(1)···O(1)	2.32	3.112(4)	153	*x*, –*y*+3/2, *z*–1/2
N(3)–H(3)···S(1)	2.69	3.546(3)	175	–*x*+2, –*y*+1, –*z*+2
N(4)–H(4)···O(1)	2.18	2.915(4)	144	*x*, –*y*+3/2, *z*–1/2
**$\mathbf{HL^6 \cdot 0.5H_2O}$**				
N(4A)–H(4)···N(2A)	2.20	2.594(3)	108	*x*, *y*, z
N(1A)–H(4)···N(2A)	2.29	2.268(4)	106	*x*, *y*, z
N(4B)–H(4)···N(2B)	2.21	2.614(5)	108	*x*, *y*, z
N(1B)–H(4)···N(2C)	2.27	2.650(3)	107	*x*, *y*, z
N(1A)–H(1)···O(1B)	2.29	3.072(3)	152	*x*, *y*, *z*
N(3A)–H(3)···S(2B)	2.77	3.600(2)	164	–*x*+2, –*y*, –*z*+2
N(4A)–H(4)···O(1B)	2.21	2.970(3)	148	*x*, *y*, *z*
N(1B)–H(1)···O(1A)	2.16	2.954(3)	153	*x*–1, *y*, *z*
N(3B)–H(3)···S(1A)	2.70	3.545(2)	169	–*x*+2, –*y*, –*z*+2
N(4B)–H(4)···O(1A)	2.19	2.972(3)	151	*x*–1, *y*, *z*
O(1*w*)–H(1)···O(2B)	1.95	2.878(6)	176	*x*, *y*, *z*
O(*w*)–H(2)···O(2A)	2.00	2.911(6)	178	*x*–1, *y*, *z*

Table S2. Crystal and Structure Refinement Data for $\mathbf{HL^2}$, $\mathbf{HL^4}$, and $\mathbf{HL^6 \cdot 0.5H_2O}$.

Compound	$\mathbf{HL^2}$	$\mathbf{HL^4}$	$\mathbf{HL^6 \cdot 0.5H_2O}$
CCDC	2517075	2517076	2517077
Empirical formula	$C_{13}H_{22}N_4O_1S_1$	$C_{13}H_{22}N_4O_1S_1$	$C_{11}H_{21}N_4O_{2.5}S_1$
Formula weight	282.40	282.40	281.38
Crystal system	triclinic	monoclinic	triclinic
Space group	$P\bar{1}$	$P2_1/c$	$P\bar{1}$
Unit cell dimensions			
a (Å)	9.4890(7)	10.8237(12)	10.1950(8)
b (Å)	10.0083(8)	16.1872(11)	10.6953(8)
c (Å)	10.0116(8)	10.0956(9)	16.0522(13)
α (°)	108.461(8)	90	73.679(7)
β (°)	117.935(8)	116.719(12)	87.488(7)
γ (°)	91.926(7)	90	63.916(8)
V (Å^3)	777.77(12)	1579.9(3)	1502.2(2)
Z	2	4	4
ρ_{calc} (g cm^{-3})	1.206	1.179	1.244
μ_{Mo} (mm^{-1})	0.207	0.204	0.221
F(000)	304	600	604
Crystal size (mm)	0.50x0.38x0.20	0.45x0.18x0.08	0.50x0.25x0.10
θ Range (°)	3.04 – 25.50	3.28 – 25.05	3.20 – 25.05
Index range	$-8 \le h \le 11$, $-10 \le k \le 12$, $-12 \le l \le 10$	$-12 \le h \le 8$, $-19 \le k \le 17$, $-12 \le l \le 11$	$-10 \le h \le 12$, $-12 \le k \le 12$, $-18 \le l \le 19$
Reflections collected / unique	4744 / 2880 (R_{int} = 0.0190)	5938 / 2783 (R_{int} = 0.0348)	9275 / 5297 (R_{int} = 0.0198)
Completeness (%)	99.6 (θ = 25.24°)	99.8 (θ = 25.05°)	99.7 (θ = 25.05°)
Reflections with $I > 2\sigma(I)$	2099	1564	3866
Number of refined parameters	173	176	344
Goodness-of-fit (GOF)	1.001	1.004	1.001
R (for $I > 2\sigma(I)$)	R_1 = 0.0578, wR_2 = 0.1635	R_1 = 0.0743, wR_2 = 0.1987	R_1 = 0.0595, wR_2 = 0.1647
R (for all reflections)	R_1 = 0.0802, wR_2 = 0.1812	R_1 = 0.1315, wR_2 = 0.2326	R_1 = 0.0846, wR_2 = 0.1858
$\Delta\rho_{max}/\Delta\rho_{min}$, ($e \cdot$Å^{-3})	0.423 / –0.294	0.425 / –0.345	0.596 / –0.406

Table S3. Selected Bond Lengths (Å) and Angles (deg) in **HL²**, **HL⁴**, and **HL⁶·0.5H₂O**

Bonds	**HL²**	**HL⁴**	**HL⁶, (A)**	**HL⁶, (B)**
	(Å)			
N(1)–C(7)	1.335(3)	1.327(4)	1.317(3)	1.328(3)
C(7)–C(8)	1.507(3)	1.495(5)	1.500(4)	1.498(4)
C(7)–O(1)	1.226(3)	1.228(4)	1.243(3)	1.239(3)
C(8)–C(9)	1.481(4)	1.495(4)	1.496(4)	1.489(4)
C(8)–N(2)	1.278(3)	1.284(4)	1.272(3)	1.282(3)
N(2)–N(3)	1.377(3)	1.357(4)	1.362(3)	1.366(3)
N(3)–C(10)	1.364(3)	1.370(4)	1.364(3)	1.374(3)
C(10)–S(1)	1.677(2)	1.672(4)	1.669(3)	1.671(3)
N(4)–C(10)	1.319(3)	1.318(5)	1.324(3)	1.326(4)
Angles	(°)			
N(1)–C(7)–C(8)	119.1(2)	115.7(3)	117.2(2)	116.7(2)
N(1)–C(7)–O(1)	124.0(2)	123.4(3)	122.9(3)	123.4(3)
C(7)–C(8)–C(9)	116.9(2)	118.6(3)	122.9(3)	117.4(2)
C(7)–C(8)–N(2)	114.5(2)	115.4(3)	115.8(2)	115.5(2)
C(9)–C(8)–N(2)	128.2(2)	115.4(3)	126.5(3)	127.1(3)
C(8)–N(2)–N(3)	119.6(2)	119.9(3)	119.4(2)	119.8(2)
N(2)–N(3)–C(10)	117.7(2)	119.9(3)	118.8(2)	119.1(2)
N(3)–C(10)–S(1)	119.6(2)	120.1(3)	119.6(2)	118.9(2)
N(4)–C(10)–S(1)	124.6(2)	125.0(3)	124.7(2)	125.4(2)
N(3)–C(10)–N(4)	115.7(2)	114.9(3)	115.7(3)	115.6(3)

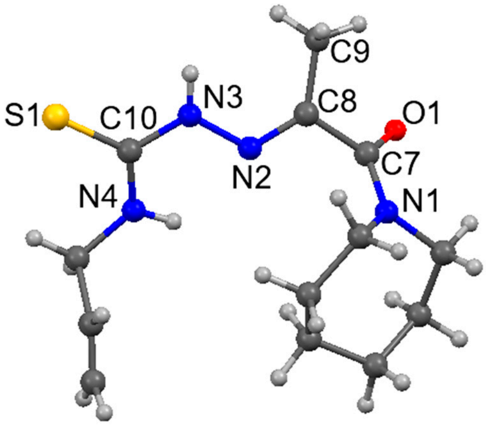

Figure S25. Molecular structure of compound **HL²** with notation of the common fragment to three structures.
